# Automated image quality assessment for selecting among multiple magnetic resonance image acquisitions in the German National Cohort study

**DOI:** 10.1038/s41598-023-49569-1

**Published:** 2023-12-20

**Authors:** Christopher Schuppert, Susanne Rospleszcz, Jochen G. Hirsch, Daniel C. Hoinkiss, Alexander Köhn, Ricarda von Krüchten, Maximilian F. Russe, Thomas Keil, Lilian Krist, Börge Schmidt, Karin B. Michels, Sabine Schipf, Hermann Brenner, Thomas J. Kröncke, Tobias Pischon, Thoralf Niendorf, Jeanette Schulz-Menger, Michael Forsting, Henry Völzke, Norbert Hosten, Robin Bülow, Maxim Zaitsev, Hans-Ulrich Kauczor, Fabian Bamberg, Matthias Günther, Christopher L. Schlett

**Affiliations:** 1 Department of Diagnostic and Interventional Radiology, Medical Center – University of Freiburg, Faculty of Medicine, University of Freiburg, Hugstetter Str. 55, 79106 Freiburg, Germany; 2 Chair of Epidemiology, Institute of Medical Information Processing, Biometry and Epidemiology, Ludwig Maximilians University, Faculty of Medicine, Munich, Germany; 3 Institute of Epidemiology, Helmholtz Zentrum München, German Research Center for Environmental Health, Neuherberg, Germany; 4 German Centre for Cardiovascular Research (DZHK), Partner Site Munich Heart Alliance, Munich, Germany; 5 Fraunhofer Institute for Digital Medicine MEVIS, Bremen, Germany; 6 Institute for Clinical Epidemiology and Biometry, University of Würzburg, Würzburg, Germany; 7 Institute of Social Medicine, Epidemiology and Health Economics, Charité – Universitätsmedizin Berlin, Berlin, Germany; 8 State Institute of Health, Bavarian Health and Food Safety Authority, Erlangen, Germany; 9 Institute for Medical Informatics, Biometry and Epidemiology, University Hospital Essen, University of Duisburg-Essen, Essen, Germany; 10 Institute for Prevention and Cancer Epidemiology, Medical Center – University of Freiburg, Faculty of Medicine, University of Freiburg, Freiburg, Germany; 11 Institute for Community Medicine, University Medicine Greifswald, Greifswald, Germany; 12 Division of Clinical Epidemiology and Aging Research, German Cancer Research Center (DKFZ), Heidelberg, Germany; 13 Division of Preventive Oncology, German Cancer Research Center (DKFZ) and National Center for Tumor Diseases (NCT), Heidelberg, Germany; 14 Department of Diagnostic and Interventional Radiology, University Hospital Augsburg, University of Augsburg, Augsburg, Germany; 15 Molecular Epidemiology Research Group, Max-Delbrueck-Center for Molecular Medicine in the Helmholtz Association (MDC), Berlin, Germany; 16 Biobank Technology Platform, Max-Delbrueck-Center for Molecular Medicine in the Helmholtz Association (MDC), Berlin, Germany; 17 Charité – Universitätsmedizin Berlin, Corporate Member of Freie Universität Berlin and Humboldt-Universität zu Berlin, Berlin, Germany; 18 Berlin Ultrahigh Field Facility (B.U.F.F.), Max Delbrück Center for Molecular Medicine in the Helmholtz Association (MDC), Berlin, Germany; 19 German Centre for Cardiovascular Research (DZHK), Partner Site Berlin, Berlin, Germany; 20 Department of Cardiology and Nephrology, HELIOS Hospital Berlin-Buch, Berlin, Germany; 21 Department of Diagnostic and Interventional Radiology and Neuroradiology, University Hospital Essen, University of Duisburg-Essen, Essen, Germany; 22 German Centre for Cardiovascular Research (DZHK), Partner Site Greifswald, University Medicine Greifswald, Greifswald, Germany; 23 Institute of Diagnostic Radiology and Neuroradiology, University Medicine Greifswald, Greifswald, Germany; 24 Division of Medical Physics, Department of Diagnostic and Interventional Radiology, Medical Center – University of Freiburg, Faculty of Medicine, University of Freiburg, Freiburg, Germany; 25 Department of Diagnostic and Interventional Radiology, Heidelberg University Hospital, Heidelberg, Germany

**Keywords:** Whole body imaging, Magnetic resonance imaging

## Abstract

In magnetic resonance imaging (MRI), the perception of substandard image quality may prompt repetition of the respective image acquisition protocol. Subsequently selecting the preferred high-quality image data from a series of acquisitions can be challenging. An automated workflow may facilitate and improve this selection. We therefore aimed to investigate the applicability of an automated image quality assessment for the prediction of the subjectively preferred image acquisition. Our analysis included data from 11,347 participants with whole-body MRI examinations performed as part of the ongoing prospective multi-center German National Cohort (NAKO) study. Trained radiologic technologists repeated any of the twelve examination protocols due to induced setup errors and/or subjectively unsatisfactory image quality and chose a preferred acquisition from the resultant series. Up to 11 quantitative image quality parameters were automatically derived from all acquisitions. Regularized regression and standard estimates of diagnostic accuracy were calculated. Controlling for setup variations in 2342 series of two or more acquisitions, technologists preferred the repetition over the initial acquisition in 1116 of 1396 series in which the initial setup was retained (79.9%, range across protocols: 73–100%). Image quality parameters then commonly showed statistically significant differences between chosen and discarded acquisitions. In regularized regression across all protocols, ‘structured noise maximum’ was the strongest predictor for the technologists’ choice, followed by ‘N/2 ghosting average’. Combinations of the automatically derived parameters provided an area under the ROC curve between 0.51 and 0.74 for the prediction of the technologists’ choice. It is concluded that automated image quality assessment can, despite considerable performance differences between protocols and anatomical regions, contribute substantially to identifying the subjective preference in a series of MRI acquisitions and thus provide effective decision support to readers.

## Introduction

Whole-body magnetic resonance imaging (MR, MRI) is a key imaging technique in population-based cohort studies, due to its excellent spatial resolution and soft-tissue contrast, its capacity for standardization, and the absence of ionizing radiation. Several studies rely on this imaging modality to generate comprehensive data repositories that can provide insights into general health and are a valuable resource for radiomics. These studies include the German National Cohort (NAKO or NAKO Health Study), the UK Biobank (UKBB), the Multi-Ethnic Study of Arteriosclerosis (MESA), the Framingham Heart Study (FHS), and the Study of Health in Pomerania (SHIP)^1,2^.

Standardized image acquisition and reproducible image quality are crucial in such studies to ensure consistent post-processing, including automated segmentation and feature extraction. MRI protocol repetitions pose a particular challenge to these objectives from a quality control standpoint due to their variable origins and presentations. Repeating MRI protocols is generally considered in case of an unsatisfactory initial image acquisition, whose quality could have been degraded for multiple reasons. Examples include, depending on the protocol in question: an improper hardware or software setup, blurring from bulk patient or organ motion, synchronization failures such as mis-triggering in the cardiac cycle, susceptibility and off-resonance artifacts, fat–water shift and swaps, anatomic coverage issues, or premature scan abortions. In a sufficiently large cohort study, these and other different quality impairments will inevitably occur, and their appearance will be multifaceted. So will be their possible remedies, if these can be determined. For quality control and imaging optimization, it is essential to deduce the root cause of quality-impaired image acquisitions, and, if the protocol was repeated, to understand the quality differences within the series of acquisitions.

The current workflow in most studies and clinical departments involves a visual assessment of all images by radiologic technologists or radiologists during or directly after acquisition. Based upon this subjective expert assessment, the respective protocol may then be repeated, possibly with updated instructions given to the subject if appropriate. The preferred image data is then chosen based on subjective assessment. This approach can be time-consuming and therefore costly, especially for large cohort studies. It also lacks standardization, is likely error-prone, and may further discomfort among participants or patients due to prolonged examinations. Computerized tools for quality control and decision support in MRI are well-suited to improve on this approach and address its shortcomings. However, existing solutions generally have a narrower focus by being confined to a specific MRI protocol, anatomical domain, or artifact type. A conceptually broader automated image quality assessment using quantitative parameters has already demonstrated the ability to predict the need for repeating an acquisition, based on the radiologic technologists’ visual assessment in the NAKO as a reference^3^. The challenge remains to automatically choose the preferred of multiple image acquisitions in a subsequent step using a comparably unrestricted approach.

Recognizing this opportunity, we primarily aimed to identify quantitative image quality parameters that can differentiate between acquisitions that were preferred and chosen by human readers as opposed to those that were discarded – yielding a prediction tool suitable for automation. A secondary objective was to learn whether MRI protocol repetitions in the NAKO improved the perceived image quality.

## Methods and materials

### Study design and population

Our project was designed as a post-hoc analysis of data from the NAKO Health Study. The NAKO is an ongoing, prospective, multicenter, population-based cohort study conducted by a network of 25 institutions at 18 regional examination sites in Germany. Its main objective is to investigate risk factors for the development of common chronic diseases such as cancer, diabetes, cardiovascular, neurodegenerative/psychiatric, respiratory, and infectious diseases^4,5^. The baseline assessment was conducted between 2014 and 2019, and 205,415 participants from the general population aged 19 to 74 years were enrolled. They received various medical and psychological assessments, including interviews, questionnaires, and physical examinations. Of these participants, 30,861 were also enrolled in the NAKO MRI study, conducted at five dedicated imaging centers. For our study, we considered all available data at the time of investigation, which comprised examinations from 11,347 participants up to December 31, 2016. This excludes examination aborts before the first MRI protocol was fully recorded or if participants withdrew consent.

The NAKO Use and Access Committee approved this study based on the participants’ informed consent, accordance with the aims of the NAKO Health Study, and ethical approval from the Ethics Committee of the Medical Faculty of the University of Heidelberg (S-843/2020). This study conformed to the ethical guidelines of the 1964 Declaration of Helsinki and its later amendments.

### MR imaging

**Figure 1:**
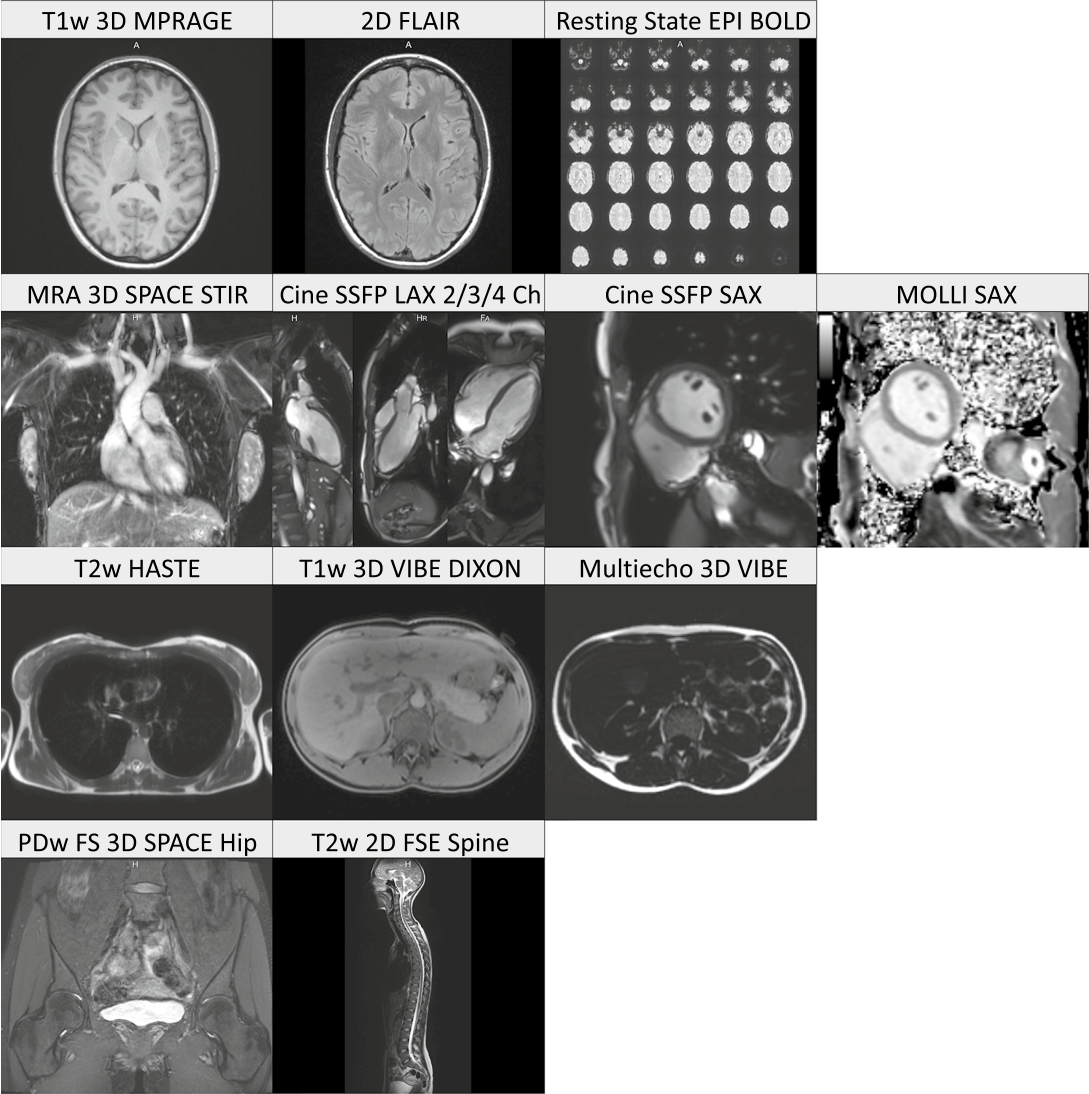
Example images from a female participant for each of the twelve protocols that were used in the baseline whole-body examination of the NAKO MRI study. Rows from top to bottom: neurodegenerative, cardiovacular, thoracoabdominal, and musculoskeletal focus group.

MRI was performed with 3T whole-body MR scanners (MAGNETOM Skyra, Siemens Healthcare, Erlangen, Germany) running an identical software version. The baseline examination program consisted of a whole-body scan with twelve protocols from four focus groups (neurodegenerative, cardiovascular, thoracoabdominal, and musculoskeletal) without intravenous contrast agent application (Fig. 1). A detailed description of the rationale, design, and technical background of the NAKO MRI study has been provided previously^1^. All image acquisitions were performed following a standard operating procedure (SOP) by radiologic technologists who were specifically trained and certified for the NAKO MRI study. The technologists were instructed to repeat a protocol if anatomic coverage did not meet the SOP, if severe image artifacts occurred, or if the image quality was unsatisfactory for other reasons. Participants were given detailed information about the scanning procedure and were instructed to move as little as possible and to follow breathing instructions.

In our analysis, we controlled for setup changes between acquisitions to obtain a subsample in which the repetitions were performed under identical technical conditions as the initial acquisitions. The following technical parameters were considered: radiofrequency (RF) coil configuration (variations in RF coils and in the selection of receive RF coil elements), field of view size, slice position (field of view shifted along the x/y/z axis of the participant), and slice orientation (field of view rotated or angled differently).

### Automated image quality assessment and choosing a preferred acquisition

**Figure 2:**
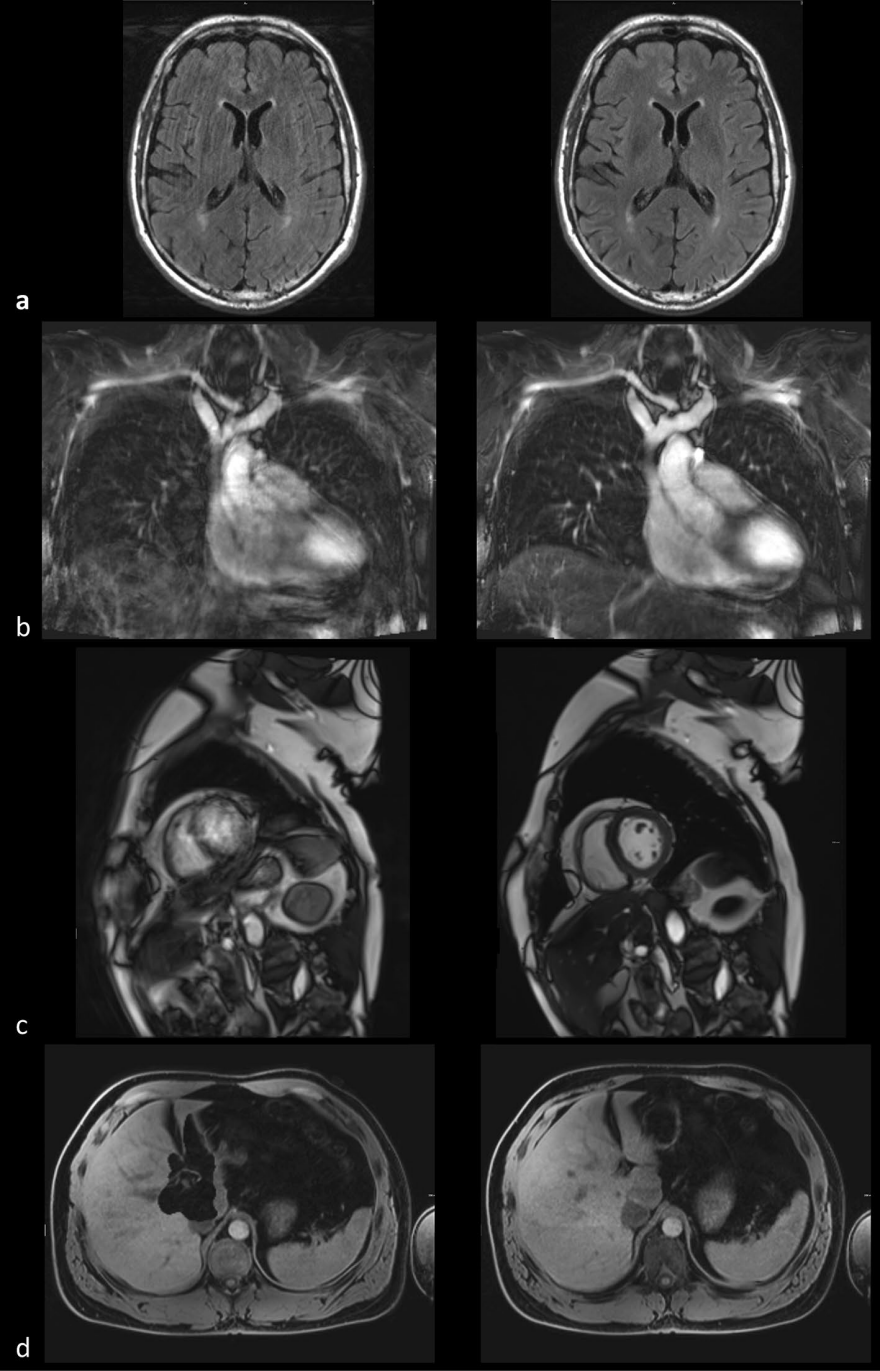
Examples of discarded (left) and chosen (right) acquisitions from four different protocols. (**a**) 2D FLAIR (considerable vs. no bulk patient motion), (**b**) MRA 3D SPACE STIR (considerable vs. moderate breathing motion), (**c**) Cine SSFP SAX (mistriggering vs. correct electrocardiographic gating), (**d**) T1w 3D VIBE DIXON (with vs. without fat–water swap artifact in the liver). The images in each pair were acquired with identical setups and taken from identical participants. In the NAKO MRI study, protocols were repeated if the initial acquisition did not meet the SOP, if severe image artifacts occurred, or if the image quality was otherwise unsatisfactory to the examining radiologic technologist.

After general data management and basic automated quality control, including verification of data completeness, conformance to predefined protocol parameters, and data uniqueness, all image series entered a processing pipeline to calculate two to eleven image(-based) quality parameters as previously reported (number varying between protocols)^3^. The results from this automated image quality assessment could be visualized through a web-based thin client that presented the image series along with the corresponding parameter values. The assessment comprised a universal quality index (‘UQI’) for general quality inspection, image sharpness, global and local signal-to-noise ratio (‘SNR’ and ‘specific SNR’), maximum and average estimates for structured image noise, maximum and average estimates of Nyquist ghosting levels (‘N/2 ghosting’), functional MRI (fMRI) signal drift and variation (‘variation over time’), and a geometric ratio between foreground and background (‘foreground ratio’). The universal quality index provides a crude, non-specific indication of image quality by considering original, noise-filtered, and edge-filtered image versions to calculate a score that increases with image noise and decreases with image blur^3^. If a protocol was repeated, the technologists defined the acquisition that they considered to be of better image quality through the thin client, as per SOP going solely by subjective image impression as long as the field of view was set correctly (Fig. 2). Only the chosen acquisition was then added to the main database for general use.

### Visual image quality rating

In the NAKO MRI study, board-certified radiologists performed a visual image quality rating for image stacks from chosen acquisitions as published previously^1^. The rating adhered to a detailed criteria catalog that considered anatomical coverage and differentiable structures along with a diverse range of potential artifacts, such as susceptibility, off-resonance, fat–water shift and swaps, banding, pulsation, and others. Scores were assigned according to a 3-point Likert scale: (1) ‘excellent’ image quality not impaired by artifacts, images appropriate for data post-processing; (2) ’good’ image quality with limited impairment by artifacts, images still appropriate for data post-processing; (3) ‘poor’ image quality due to artifacts or insufficient coverage, images generally not appropriate for post-processing. The protocols used for functional or quantitative imaging (Resting State EPI BOLD, MOLLI SAX, and Multiecho 3D VIBE) were not rated. The criteria catalog is shown in Supplemental Material Table S1. For our analysis, we examined the differences in quantitative image quality parameters between the visual quality ratings while, in the same manner as described above, controlling for setup changes between initial acquisitions and repetitions.

### Statistics

Data on participants, acquisitions, and repetitions are presented as counts and percentages.

Given repetitions with the same setup as the initial acquisition, the mean differences in image quality parameters between discarded acquisitions and chosen acquisitions were assessed by paired t-tests or signed rank tests after testing for normality using Shapiro–Wilk tests. The mean differences in image quality parameters between the ordinal visual quality ratings were assessed by Kruskal–Wallis tests and limited to parameters that were available for > 70% of the acquisitions in the relevant subsample (‘UQI’, ‘SNR’, ‘sharpness’, and ‘foreground ratio’) to achieve sufficient statistical power.

Associations of single image quality parameters with outcome chosen vs. discarded were evaluated by logistic regression models providing odds ratios (OR) with corresponding 95% confidence intervals (CI) per standard deviation of image quality parameters. To investigate the effect of inter-individual variation, generalized linear mixed models with logit link and random intercept per participant were calculated.

Discriminative performance of the combined set of image quality parameters to distinguish between chosen and discarded acquisitions was assessed by regularized logistic regression with an elastic net penalty and hyperparameters computed by fivefold cross-validation. We calculated three models with alpha values corresponding to LASSO regression, ridge regression, and a balanced blend thereof (Elastic Net Regression). All models were run on 1000 bootstrap samples of the data to quantify the relative importance of the respective image quality parameters by their selection frequency over all samples. Area under the Receiver Operating Characteristic (ROC) curve (AUC) served as the measure of discriminative performance.

Analyses were conducted for all protocols combined, as well as stratified by protocol. P-values < 0.05 were considered to indicate statistical significance. SAS version 9.4 and R version 4.1 were used for all analyses.

## Results

In 1,359 (12.0%) of the 11,347 participants, one or more initial acquisitions of a protocol were followed by at least one repetition. With these included, 135,845 acquisitions were performed, of which 134,239 (98.8%) were initial acquisitions and 1606 (1.2%) were repetitions. Most repetitions were limited to one with 1558 (1.2%) first repetitions, 46 (0.03%) second repetitions, and 2 (0.001%) third repetitions. Table 1 details repetition frequencies on a per-protocol level. Of all repetitions, 807 (50.2%) retained the setup of their respective initial acquisition with no changes to RF coil configuration, FOV size, slice position, or slice orientation. These parameters were changed in 81 (5.0%), 53 (3.3%), 791 (49.3%), and 203 (12.6%) repetitions.

**Table 1:** Overview of repetitions by protocol.

Protocol	All repetitions	1st repetitions	2nd repetitions	3rd repetitions	Repetitions with an identical setup as the initial acquisition (% of all repetitions)
All	1606	1558	46	2	807 (50.2)
T1w 3D MPRAGE	114	114			87 (76.3)
2D FLAIR	335	326	9		191 (57.0)
Resting State EPI BOLD	7	7			1 (14.3)
MRA 3D SPACE STIR	51	51			20 (39.2)
Cine SSFP LAX	417	392	23	2	322 (77.2)
Cine SSFP SAX	115	115			63 (54.8)
MOLLI SAX	11	11			6 (54.5)
T2w HASTE	83	82	1		30 (36.1)
T1w 3D VIBE Dixon	98	94	4		53 (54.1)
Multiecho 3D VIBE	225	216	9		30 (13.3)
PDw FS 3D SPACE	117	117			2 (1.7)
T2w 2D FSE	33	33			2 (6.1)

Columns two to five (all, 1st, 2nd, and 3rd repetitions) consider repetitions regardless of setup changes.

The technologists were asked to choose a preferred acquisition for 2,342 series of initial acquisitions and repetitions. This is more than the underlying 1,558 initial acquisitions shown in Table 1 due to the individual assessment of the three orientations 2Ch, 3Ch, and 4Ch in the Cine SSFP LAX protocol used for cardiac imaging. The sample size decreased to 1,396 (59.6%) when limited to repetitions that retained the initial setup. For this particular subsample, the technologists chose the repetition over the initial acquisition in 1,116 (79.9%) instances, with a range of 73%-100% across protocols (Table 2). Considering all acquisitions without controlling for setup variations, this share increased to 90.4%.

**Table 2:** The summed series of initial acquisitions and repetitions the radiologic technologists assessed to choose a preferred acquisition; overall (n = 2342) and limited to repetitions that retained the initial setup (n = 1396). Repetitions were preferably chosen over initial acquisitions across all protocols (range: 73–100%).

Protocol	Series of initial acquisitions and repetitions, overall	Series of initial acquisitions and repetitions, limited to repetitions that retained the initial setup	Repetition chosen of the column to the left (%)
All	2342	1396	1116 (79.9)
T1w 3D MPRAGE	114	87	81 (93.1)
2D FLAIR	326	191	174 (91.1)
Resting State EPI BOLD	7	1	1 (100)
MRA 3D SPACE STIR	51	20	17 (85.0)
Cine SSFP LAX 2Ch	392	303	222 (73.3)
Cine SSFP LAX 3Ch	392	306	224 (73.2)
Cine SSFP LAX 4Ch	392	305	236 (77.4)
Cine SSFP SAX	115	63	53 (84.1)
MOLLI SAX	11	6	6 (100)
T2w HASTE	82	29	26 (89.7)
T1w 3D VIBE Dixon	94	52	47 (90.4)
Multiecho 3D VIBE	216	29	25 (86.2)
PDw FS 3D SPACE	117	2	2 (100)
T2w 2D FSE	33	2	2 (100)

### Prediction of the technologists’ choice based on the quantitative image quality parameters

In an analysis limited to series where all repetitions retained the setup of the initial acquisition, the quantitative image quality parameters largely showed significant differences between chosen and discarded acquisitions when considering all protocols (Table 3). Only the parameter ‘UQI’ did not differentiate with statistical significance for this subsample, for which the parameters ‘drift’ and ‘variation over time’ were not tested due to insufficient sample size. Testing stratified by protocol revealed that differences were predominantly significant for protocols from the neurodegenerative and cardiac focus groups, which were also the ones with the largest sample sizes.

**Table 3:** Statistical significances of the absolute differences in image quality parameters between chosen and discarded acquisitions from zero.

Protocol	UQI	Sharpness	SNR	Specific SNR	Structured noise max	Structured noise avg	N/2 ghosting max	N/2 ghosting avg	Drift	Variation over time	Foreground rati**o**
All	0.86 1396	** < 0.001** 1396	**0.003** 1374	** < 0.001** 281	** < 0.001** 796	** < 0.001** 796	** < 0.001** 808	** < 0.001** 808	- 1	- 1	**0.04** 1374
T1w 3D MPRAGE	**0.002** 87	** < 0.001** 87	** < 0.001** 87	0.83 87	**0.001** 87	** < 0.001** 87	0.17 87	0.06 87	NA	NA	0.18 87
2D FLAIR	** < 0.001** 191	** < 0.001** 191	** < 0.001** 191	** < 0.001** 191	** < 0.001** 191	** < 0.001** 191	** < 0.001** 191	** < 0.001** 191	NA	NA	** < 0.001** 191
Resting state EPI BOLD	- 1	- 1	- 1	- 1	- 1	- 1	- 1	- 1	- 1	- 1	- 1
MRA 3D SPACE STIR	0.79 20	0.93 20	NA	NA	NA	NA	NA	NA	NA	NA	NA
Cine SSFP LAX 2Ch	0.24 303	0.16 303	0.90 303	NA	0.47 14	0.47 14	0.50 16	0.50 16	NA	NA	0.90 303
Cine SSFP LAX 3Ch	0.32 306	** < 0.001** 306	**0.004** 306	NA	** < 0.001** 264	** < 0.001** 264	** < 0.001** 267	** < 0.001** 267	NA	NA	**0.002** 306
Cine SSFP LAX 4Ch	0.08 305	** < 0.001** 305	0.67 305	NA	0.30 71	0.30 71	0.88 79	0.88 79	NA	NA	0.65 305
Cine SSFP SAX	**0.01** 63	**0.008** 63	0.42 63	NA	**0.04** 59	**0.03** 59	0.07 59	**0.006** 59	NA	NA	0.49 63
MOLLI	0.15 6	**0.03** 6	0.64 6	NA	- 2	- 2	- 1	- 1	NA	NA	0.37 6
T2w HASTE	0.60 29	0.39 29	0.70 29	NA	0.33 29	0.71 29	0.18 29	0.48 29	NA	NA	**0.03** 29
T1w 3D VIBE DIXON	**0.04** 52	0.15 52	0.38 52	NA	0.14 52	0.76 52	0.06 52	0.96 52	NA	NA	0.14 52
Multiecho 3D VIBE	**0.001** 29	0.25 29	0.12 29	NA	0.93 24	0.76 24	0.66 24	0.18 24	NA	NA	**0.03** 29
PDw FS 3D SPACE	- 2	- 2	NA	NA	NA	NA	NA	NA	NA	NA	NA
T2w 2D FSE	- 2	- 2	- 2	- 2	- 2	- 2	- 2	- 2	NA	NA	- 2

P-values (upper row) and number of comparisons (lower row) are from a paired t-test or signed rank test depending on normality. Note that this table considers only repetitions that retained the setup of the initial acquisition, and compares only initial acquisitions to repetitions, not repetitions to repetitions – therefore and because, for technical reasons, the quantitative image quality parameters were not derived from every acquisition, the number of comparisons may differ from the underlying series of initial acquisitions and repetitions shown in Table 1.

Bold denotes statistical significance at p < 0.05. *NA* not available.

In logistic regression, the single image quality parameters showed varying associations with the outcome chosen vs. discarded (Table 4). The highest observed OR was 2.53 (p = 0.23) for ‘sharpness’ in the MOLLI protocol. In generalized linear mixed models, an effect of inter-individual variation could not be established (variance of random effects equal to zero).

**Table 4:** Associations of single image quality parameters with the outcome ‘chosen vs. discarded acquisition’.

Protocol	UQI			Sharpness			SNR			Specific SNR					
	OR	CI	p-value	OR	CI	p-value	OR	CI	p-value	OR	CI	p-value			
All	1	[0.93, 1.07]	0.93	1.04	[0.97, 1.12]	0.27	1.03	[0.96, 1.11]	0.39	1.16	[0.98, 1.37]	0.08			
T1w 3D MPRAGE	0.8	[0.57, 1.08]	0.15	1.74	[1.26, 2.44]	0.001	1.6	[1.17, 2.22]	0.004	1.11	[0.82, 1.52]	0.49			
2D FLAIR	0.89	[0.73, 1.09]	0.27	1.37	[1.12, 1.7]	0.003	2.52	[1.98, 3.27]	< 0.001	1.9	[1.52, 2.4]	0			
Resting State EPI BOLD	NA	NA	NA	NA	NA	NA	NA	NA	NA	NA	NA	NA			
MRA 3D SPACE STIR	1.04	[0.55, 2.01]	0.9	1.1	[0.58, 2.1]	0.77	NA	NA	NA	NA	NA	NA			
Cine SSFP LAX 2Ch	0.98	[0.84, 1.15]	0.84	1.01	[0.86, 1.19]	0.89	0.97	[0.82, 1.13]	0.68	NA	NA	NA			
Cine SSFP LAX 3Ch	0.99	[0.84, 1.16]	0.87	1.1	[0.94, 1.29]	0.24	1.24	[1.05, 1.47]	0.01	NA	NA	NA			
Cine SSFP LAX 4Ch	1.02	[0.87, 1.2]	0.82	1.08	[0.92, 1.27]	0.32	1	[0.85, 1.17]	0.96	NA	NA	NA			
Cine SSFP SAX	1.06	[0.74, 1.51]	0.76	1.15	[0.81, 1.64]	0.45	1.09	[0.77, 1.57]	0.63	NA	NA	NA			
MOLLI	1.7	[0.5, 8.58]	0.42	2.53	[0.69, 18.46]	0.23	1.15	[0.34, 4.23]	0.81	NA	NA	NA			
T2w HASTE	1.01	[0.68, 1.49]	0.97	1.03	[0.7, 1.53]	0.87	0.98	[0.67, 1.45]	0.93	NA	NA	NA			
T1w 3D VIBE DIXON	1	[0.59, 1.7]	0.99	1.01	[0.6, 1.72]	0.96	1.05	[0.62, 1.77]	0.87	NA	NA	NA			
Multiecho 3D VIBE	0.79	[0.43, 1.34]	0.39	1.05	[0.62, 1.78]	0.87	0.94	[0.55, 1.58]	0.80	NA	NA	NA			
PDw FS 3D SPACE	NA	NA	NA	NA	NA	NA	NA	NA	NA	NA	NA	NA			
T2w 2D FSE	NA	NA	NA	NA	NA	NA	NA	NA	NA	NA	NA	NA			

Protocol	Structured noise max			Structured noise avg			N/2 ghosting max			N/2 ghosting avg			Foreground ratio		
	OR	CI	p-value	OR	CI	p-value	OR	CI	p-value	OR	CI	p-value	OR	CI	p-value
All	0.77	[0.7, 0.85]	< 0.001	0.81	[0.73, 0.89]	< 0.001	0.84	[0.76, 0.93]	< 0.001	0.8	[0.72, 0.88]	< 0.001	1	[0.93, 1.08]	0.92
T1w 3D MPRAGE	0.69	[0.5, 0.94]	0.02	0.73	[0.53, 0.99]	0.047	0.86	[0.64, 1.16]	0.33	0.9	[0.66, 1.21]	0.49	0.9	[0.66, 1.21]	0.49
2D FLAIR	0.55	[0.44, 0.69]	< 0.001	0.55	[0.43, 0.68]	< 0.001	0.54	[0.43, 0.67]	< 0.001	0.58	[0.46, 0.72]	< 0.001	1.07	[0.87, 1.31]	0.52
Resting State EPI BOLD	NA	NA	NA	NA	NA	NA	NA	NA	NA	NA	NA	NA	NA	NA	NA
MRA 3D SPACE STIR	NA	NA	NA	NA	NA	NA	NA	NA	NA	NA	NA	NA	NA	NA	NA
Cine SSFP LAX 2Ch	0.89	[0.48, 1.6]	0.69	0.89	[0.48, 1.6]	0.69	0.64	[0.34, 1.12]	0.13	0.64	[0.34, 1.12]	0.13	0.98	[0.83, 1.15]	0.77
Cine SSFP LAX 3Ch	0.75	[0.63, 0.89]	0.001	0.75	[0.63, 0.89]	0.001	0.77	[0.64, 0.91]	0.003	0.77	[0.64, 0.91]	0.003	1.09	[0.93, 1.29]	0.27
Cine SSFP LAX 4Ch	0.93	[0.68, 1.27]	0.66	0.93	[0.68, 1.27]	0.66	0.84	[0.61, 1.12]	0.24	0.84	[0.61, 1.12]	0.24	1.03	[0.88, 1.21]	0.71
Cine SSFP SAX	0.73	[0.5, 1.06]	0.10	0.77	[0.52, 1.1]	0.16	0.83	[0.57, 1.19]	0.32	0.81	[0.56, 1.16]	0.25	1	[0.7, 1.43]	0.99
MOLLI	NA	NA	NA	NA	NA	NA	NA	NA	NA	NA	NA	NA	1.18	[0.35, 4.19]	0.79
T2w HASTE	1.17	[0.79, 1.74]	0.44	1.03	[0.7, 1.53]	0.87	1.23	[0.84, 1.85]	0.29	1.08	[0.73, 1.6]	0.70	0.96	[0.65, 1.42]	0.85
T1w 3D VIBE DIXON	0.82	[0.47, 1.38]	0.46	0.88	[0.51, 1.49]	0.64	0.89	[0.52, 1.51]	0.67	0.93	[0.55, 1.57]	0.78	0.94	[0.55, 1.58]	0.80
Multiecho 3D VIBE	0.92	[0.51, 1.63]	0.77	0.96	[0.54, 1.71]	0.90	0.95	[0.53, 1.67]	0.84	1.04	[0.58, 1.87]	0.90	0.93	[0.55, 1.57]	0.78
PDw FS 3D SPACE	NA	NA	NA	NA	NA	NA	NA	NA	NA	NA	NA	NA	NA	NA	NA
T2w 2D FSE	NA	NA	NA	NA	NA	NA	NA	NA	NA	NA	NA	NA	NA	NA	NA

Odds ratios (OR) with corresponding 95% confidence interval (CI) and p-value taken from a logistic regression model. ORs are given per standard deviation of the image quality parameter per protocol. The protocols Resting State EPI BOLD, PDw FS 3D SPACE, and T2w 2D FSE as well as the parameters ‘drift’ and ‘variation over time’ had an insufficient sample size for inclusion.

**Figure 3:**
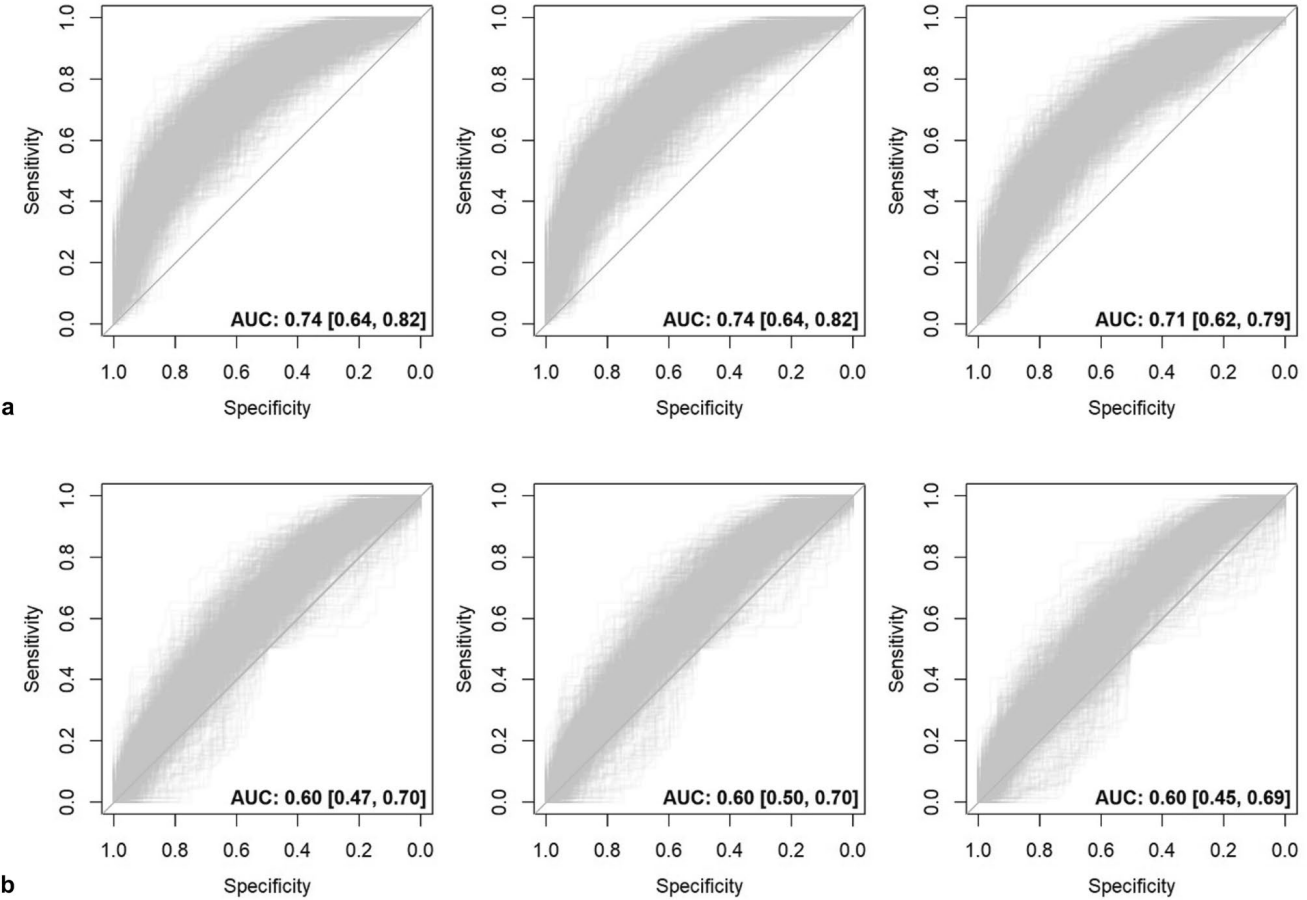
ROC curves from regularized regression of the combined set of image quality parameters with the outcome ‘chosen vs. discarded acquisition’ for two protocol examples: (**a**) T1w 3D MPRAGE (above-average performance), (**b**) Cine SSFP LAX 4Ch (below-average performance). AUC with 95% CI corresponds to mean AUC and respective percentiles from the distribution over all bootstrap samples. Left to right: LASSO regression, Elastic net regression, ridge regression.

**Figure 4:**
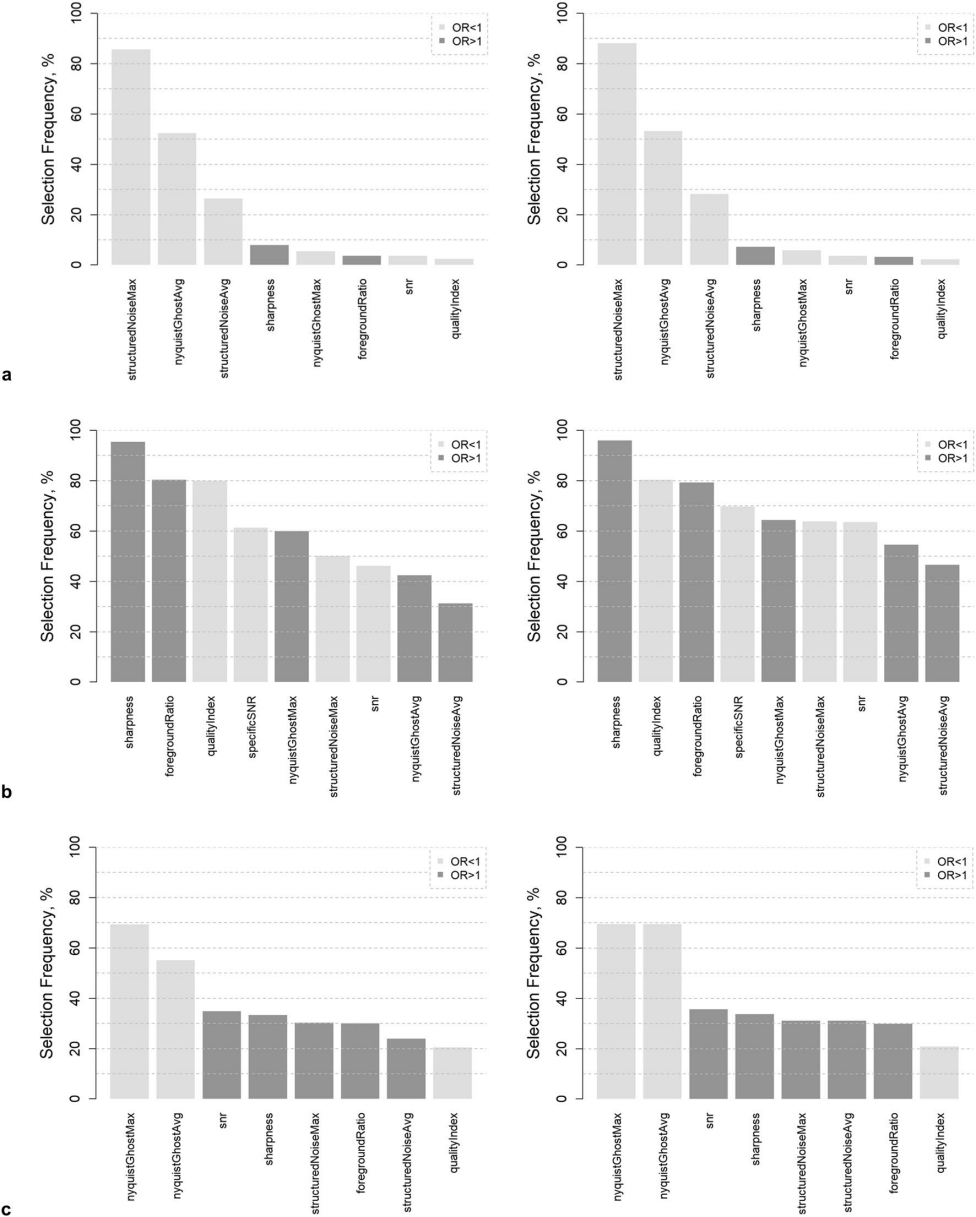
Variable selection frequencies from regularized regression with the outcome ‘chosen vs. discarded acquisition: (**a**) across all protocols on 1000 bootstrap samples*, (**b**) for T1w 3D MPRAGE (above-average performance), (**c**) for Cine SSFP LAX 4Ch (below-average performance). Left: LASSO regression, right: Elastic Net regression. As there is no variable selection in ridge regression, all selection frequencies are 100% (therefore not shown). *To minimize skewing towards protocols from the neurodegenerative focus group due to missing data, the parameter ‘specific SNR’ was excluded from potential predictors.

In regularized regression of the combined set of image quality parameters with the outcome chosen vs. discarded (Table 5), the discriminative performance was low if considering all protocols (excluding the parameter ‘specific SNR’ to minimize missing data) with an AUC of 0.58 (95% CI 0.56, 0.61) for LASSO regression as well as Elastic Net regression, and a similarly low AUC of 0.59 (95% CI 0.56, 0.61) for ridge regression. Stratified by protocol, however, the AUC varied considerably between 0.51 and 0.74, with the best discriminative performance for two protocols from the neurodegenerative focus group; T1w 3D MPRAGE with AUC 0.74 (95% CI 0.64, 0.82) and 2D FLAIR with AUC 0.73 (95% CI 0.68, 0.78), again identical for LASSO regression and Elastic Net regression and only slightly different for ridge regression (Fig. 3, Supplemental Material Fig. S1). Selection frequencies across all protocols on 1000 bootstrap samples (again excluding ‘specific SNR’) showed that the most relevant parameter for distinguishing chosen and discarded acquisitions was the maximum value of ‘structured noise’, followed by the average of ‘N/2 ghosting’. The strongest predictors also differed across the individual protocols (Fig. 4, Supplemental Material: Fig. S2).

**Table 5:** AUC with 95% CI from regularized regression of the combined set of image quality parameters with the outcome ‘chosen vs. discarded acquisition’.

Protocol	LASSO	Elastic net	Ridge
All protocols	0.69 [0.64, 0.73]	0.69 [0.64, 0.73]	0.67 [0.63, 0.72]
All protocols ('specific SNR’ excluded)*	0.58 [0.56, 0.61]	0.58 [0.56, 0.61]	0.59 [0.56, 0.61]
T1w 3D MPRAGE	0.74 [0.64, 0.82]	0.74 [0.64, 0.82]	0.71 [0.62, 0.79]
2D FLAIR	0.73 [0.68, 0.78]	0.73 [0.68, 0.78]	0.72 [0.67, 0.77]
Resting State EPI BOLD	NA	NA	NA
MRA 3D SPACE STIR	0.51 [0.50, 0.71]	0.51 [0.50, 0.72]	0.52 [0.50, 0.72]
Cine SSFP LAX 2Ch	0.57 [0.50, 0.86]	0.57 [0.50, 0.87]	0.58 [0.50, 0.86]
Cine SSFP LAX 3Ch	0.60 [0.55, 0.64]	0.60 [0.55, 0.64]	0.60 [0.56, 0.64]
Cine SSFP LAX 4Ch	0.60 [0.47, 0.70]	0.60 [0.50, 0.70]	0.60 [0.45, 0.69]
Cine SSFP SAX	0.63 [0.50, 0.74]	0.63 [0.50, 0.74]	0.63 [0.50, 0.72]
MOLLI	0.68 [0.50, 1.00]	0.69 [0.50, 1.00]	0.71 [0.50, 1.00]
T2w HASTE	0.53 [0.50, 0.78]	0.53 [0.50, 0.79]	0.53 [0.50, 0.78]
T1w 3D VIBE DIXON	0.62 [0.50, 0.72]	0.61 [0.50, 0.72]	0.61 [0.50, 0.71]
Multiecho 3D VIBE	0.54 [0.50, 0.83]	0.54 [0.50, 0.83]	0.54 [0.50, 0.82]
PDw FS 3D SPACE	NA	NA	NA
T2w 2D FSE	NA	NA	NA

The given values correspond to mean AUC and respective percentiles from the distribution over all bootstrap samples or individual protocols. Three protocols had an insufficient sample size for inclusion: Resting State EPI BOLD, PDw FS 3D SPACE, and T2w 2D FSE.

*To minimize skewing towards protocols from the neurodegenerative focus group due to missing data, the parameter ‘specific SNR’ was excluded from potential predictors.

### Differences in the quantitative image quality parameters between visual quality ratings

**Figure 5:**
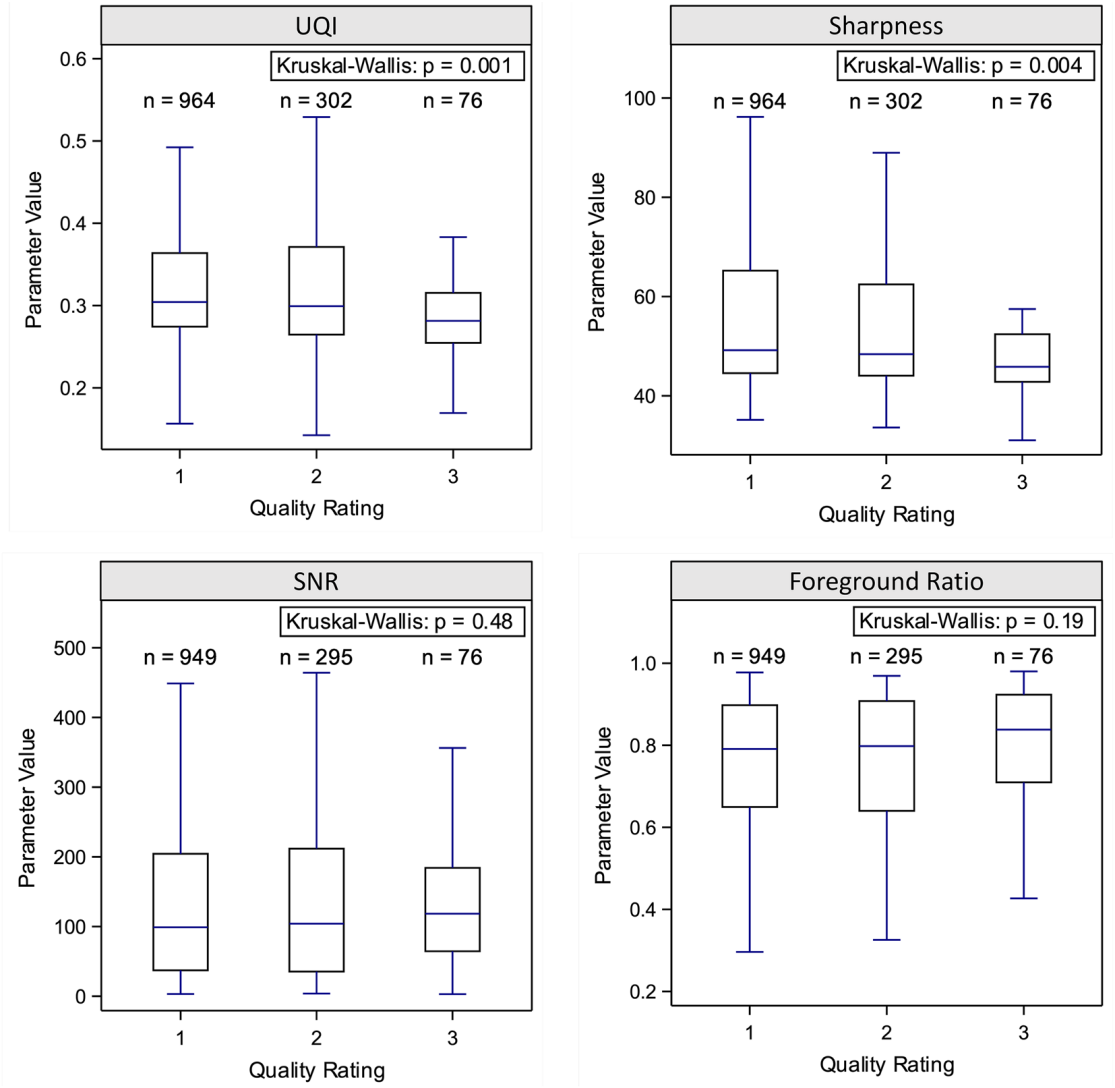
Box plots and p-values from Kruskal–Wallis tests for quantitative image quality parameters grouped by visual quality ratings of the image stacks from chosen acquisitions. The visual quality rating followed objective criteria to assign a score based on a 3-point Likert scale (1: ‘excellent’, 2: ‘good’, 3: ‘poor’). Each plot considers all protocols for which the respective parameter was calculated. *UQI* universal quality index, *SNR* signal-to-noise ratio.

In the analyzed subsample of chosen acquisitions, visual quality ratings were available for 97.2% of the corresponding image stacks. Across all protocols, the radiologists rated 71.8% of the image stacks as ‘excellent’, 22.5% as ‘good’, and 5.7% as ‘poor’. The prevalence of the ‘poor’ rating ranged from 0 to 14.3% across protocols. Significance differences between the visual quality ratings were observed for the parameters ‘UQI’, ‘sharpness’, and ‘foreground ratio’, leading to decreased values of ‘UQI’ and ‘sharpness’ in lower rated and increased values of ‘foreground ratio’ in higher rated acquisitions. No significant differences were observed for ‘SNR’ (Fig. 5). The AUC for identifying ‘poor’ acquisitions from the combined parameters ranged from 0.61 (protocol: Cine SSFP LAX 2Ch) to 0.98 (protocol: T1w 3D MPRAGE).

## Discussion

Our analysis of the NAKO MRI study, comprising 11,347 whole-body examinations with 135,845 acquisitions using twelve protocols, showed that 134,239 (98.8%) were initial acquisitions and 1606 (1.2%) were repetitions. In a subsample limited to repetitions that retained the initial setup, radiologic technologists comparatively assessed 1396 series of initial acquisitions and repetitions to determine the highest-quality images, and by a rate of 79.9% chose a repetition. An automated image quality assessment demonstrated varying classification abilities for this task, with areas under the receiver operating characteristic curve (AUC) between 0.51 and 0.74, depending on parameter selection and protocol.

Existing approaches for the automation of quality control in MRI have a narrower focus by being confined to a specific MRI protocol, anatomical domain, or artifact type: Esteban et al. trained a random forests classifier on 1101 T1w brains scans of a multi-site dataset to predict a binary quality label (‘accept’ or ‘exclude’) from 14 image quality metrics^6^. Alexander-Bloch et al. used an estimate of microscopic head motion on fMRI time series as a proxy measure for motion during a preceding same-subject T1w acquisition^7^. Earlier studies on the mitigation of motion artifacts used optical tracking systems to measure and also prospectively correct microscopic head motion^8^. Ahmad et al. recently adapted a convolutional neural network to classify diffusion-weighted brain images into artifactual and non-artifactual based on motion-induced signal dropout, interslice instability, ghosting, chemical shift, and susceptibility^9^. Aside from neuroimaging, Tarroni et al. developed an extensive quality control pipeline for cardiac cine short-axis stacks from the UK Biobank considering heart coverage, inter-slice motion, and image contrast^10,11^. Other published approaches, while notably effective for a specific task, were similarly restricted. In our previous investigation of automated image quality assessment in the NAKO MRI study, we used a broader approach and found that the eleven parameters also used in the present study distinguished initial acquisitions that were seen necessary to repeat from those that were not with varying performance between protocols: Using different parameter combinations not narrowed to a specific artifact type (for a subsample again limited to acquisitions that were not associated with subsequent setup changes), the AUC ranged from 0.58 to 0.99, or up to 0.89 after removal of a debatable outlier^3^. The discriminative performance in that previous investigation was highest for protocols from the neurodegenerative focus group. Our current study extends this broad approach to the differentiation of chosen and discarded acquisitions on an intra-participant level. The overall slightly lower performance suggests less pronounced image quality differences in these relative to repeated and not repeated initial acquisitions that were compared on an inter-participant level. This is supported by the possibility that improvements in the same participant were not necessarily achieved, whereas initial acquisitions only qualified for repetition if they were noticeably different from the overall set of high-quality acquisitions. It is, however, somewhat contradicted by the 79.9% choice rate for repetitions, which does suggest obvious quality differences. But this rate may also be partially biased by the choice procedure, which was unblinded to the acquisition order (initial or repetition) as well as the quality parameters, even if those were not to be considered as per SOP. The performance of the regression models may have been better if chosen and discarded acquisitions with setup changes (such as variations in RF coils) and presumably starker image quality differences had been included. Yet for these, the choice was not one of preference but of adherence to the SOP and an automatic image quality assessment for decision support therefore inconsequential.

The discriminative performance of our approach varied considerably between the protocols, and different parameters or their combinations worked superiorly on some protocols compared to others. While the distinct physical properties of each parameter and their relevance for a certain protocol will be partly responsible for this, there are additional factors contributing to performance variations: In certain protocols, especially those with a cardiovascular focus, the diagnostically relevant image region is substantially smaller than the overall field of view. A parameter that is averaged across the entire three- or four-dimensional image stack may then, while technically correct, give a skewed representation of the images’ usability. Examples for the Cine SSFP SAX and LAX protocols are the presence of cardiac motion artifacts that typically arise from ineffective electrocardiographic gating (‘mistriggering’) or banding artifacts from off-resonance effects. The reverse may also apply: Low-quality image areas can degrade a parameter score without significantly degrading the images’ usability. For instance, in a T1w 3D MPRAGE protocol, organ motion artifacts from swallowing will negatively influence the overall image sharpness without affecting the depiction of intracranial structures. A solution to this problem could be the regional localization of image quality parameters through the implementation of bounding boxes for areas of interest or through organ segmentations. A similar point was made by Esteban et al.^6^. To preserve the automatic workflow, however, this would necessitate the automatic creation of such delineations. Appropriate segmentation algorithms are already available and continuously improving with further advances in machine learning^12–14^. Our approach could also be complemented by other automatic quality assessment techniques that assess the images’ metadata, perform cross-correlations between protocols, or investigate recreated k-spaces. In the detection of cardiac motion artifacts, for example, a k-space approach tested on UK Biobank data achieved an excellent classification performance with an AUC of 0.89^15^. Implemented into scan assistant software, by itself or as one element of a wider quality control pipeline, such an enhanced approach would likely optimize throughput in large cohort studies, screening programs, or clinical imaging by minimizing the need for human intervention. The accompanying image quality harmonization would benefit downstream post-processing algorithms that rely on consistently high image quality for segmentation tasks or computer-aided diagnosis. In its current form, however, our approach is best applicable to the protocols T1w 3D MPRAGE and 2D FLAIR based on their respective AUC values in the regression models and has considerably less value outside neuroimaging. If it can be augmented with the additional techniques described above, further usability seems plausible especially for protocols employed in cardiac imaging, which showed the next highest AUC values in the present study. Nonetheless, it may have to be discarded completely for some protocols.

A strength of this study is that it draws from a large database of MRI examinations that were performed in a highly controlled setting. The following image quality assessment was strictly standardized through its automated methodology, independent of specialized hardware such as phantoms or sensors, and not constricted to a specific MRI protocol or artifact type. A limitation is the aforementioned bias resulting from unblinded choices. Another important limitation is that we were unable to measure the differences in quality parameters between chosen and discarded acquisitions against the intra-participant intra-protocol variabilities in multiple satisfactory acquisitions since repeated measurements were only performed after an unsatisfactory initial acquisition and only one was then chosen. A further limitation is that the radiologic technologists were able to brief participants before performing a repetition: By reminding them to follow breathing instructions, cease motion, or otherwise better comply with the examination, the technologists introduced a selection bias and put the repetitions to an advantage, as was their responsibility to optimize imaging. Independent from these limitations, we showed that several quantitative image quality parameters also differed statistically significantly in mean values between the ‘poor’, ‘good’, and ‘excellent’ visual quality ratings assigned by board-certified radiologists, providing further evidence of an association between the parameters and the visual quality impression as evaluated with objective criteria. Our statistical analysis is constrained by the use of a diagnostic prediction model on a known dataset, as opposed to a prognostic prediction model on unknown data. The weak points listed above could be addressed by conducting further examinations in a blinded and controlled manner. The subsample analysis of repetitions without setup changes was restricted by particularly low counts for the protocols Resting State EPI BOLD (n = 1), PDw FS 3D SPACE (n = 2), T2 2D FSE (n = 2), and MOLLI SAX (n = 6), which meant the exclusion of the first three. A constraint inherent to the image quality parameters is interdependences between individual and compound parameters, such as ‘noise’ and ‘signal-to-noise ratio’. Lastly, as was the case in our previous study, the ability of this automated image quality assessment to generalize on clinical data with inherently less standardization has yet to be validated.

In conclusion, our approach for automated image quality assessment can, despite varying accuracy for different protocols and anatomical regions, contribute substantially to identifying the subjective preference in a series of MRI acquisitions and thus provide effective decision support to readers in large-scale imaging studies and potentially in clinical imaging.

### Supplementary Information

Supplementary Information.

## Data Availability

The data generated and/or analyzed during the current study are available upon request from the data transfer unit of the NAKO Health Study (https://transfer.nako.de or transfer@nako.de). All data requests and project agreements are subject to approval by the NAKO Health Study board.

## Abbreviations

AUC: Area under the curve
CI: Confidence interval
FHS: Framingham heart study
fMRI: Functional MRI
MESA: Multi-ethnic study of arteriosclerosis
MR: Magnetic resonance
MRI: Magnetic resonance imaging
NAKO: German National Cohort / NAKO Health Study
OR: Odds ratio
ROC: Receiver operating characteristic
RF: Radiofrequency (coil)
SHIP: Study of Health in Pomerania
UKBB: UK Biobank
SNR: Signal-to-noise ratio
SOP: Standard operating procedure
UQI: Universal quality index
