# Supplementary material for: Automated image quality assessment for selecting among multiple magnetic resonance image acquisitions in the German National Cohort study
Source: Sci Rep. 2023 Dec 20;13:22745. doi: 10.1038/s41598-023-49569-1 (PMC10733361; doi:10.1038/s41598-023-49569-1)

Automated Image Quality Assessment for Selecting Among Multiple Magnetic Resonance Image Acquisitions in the German National Cohort Study

Christopher Schuppert[1], Susanne Rospleszcz[2,3,4], Jochen G. Hirsch[5], Daniel C. Hoinkiss[5], Alexander Köhn[5], Ricarda von Krüchten[1], Maximilian F. Russe[1], Thomas Keil[6,7,8], Lilian Krist[7], Börge Schmidt[9], Karin B. Michels[10], Sabine Schipf[11], Hermann Brenner[12,13], Thomas J. Kröncke[14], Tobias Pischon[15,16,17], Thoralf Niendorf[18], Jeanette Schulz-Menger[17,19,20], Michael Forsting[21], Henry Völzke[11,22], Norbert Hosten[23], Robin Bülow[23], Maxim Zaitsev[24], Hans-Ulrich Kauczor[25], Fabian Bamberg[1], Matthias Günther[5], Christopher L. Schlett[1]

[1]Department of Diagnostic and Interventional Radiology, Medical Center – University of Freiburg, Faculty of Medicine, University of Freiburg, Freiburg, Germany

[2]Chair of Epidemiology, Institute of Medical Information Processing, Biometry and Epidemiology, Ludwig Maximilians University, Faculty of Medicine, Munich, Germany

[3]Institute of Epidemiology, Helmholtz Zentrum München, German Research Center for Environmental Health, Neuherberg, Germany

[4]German Centre for Cardiovascular Research (DZHK), Partner Site Munich Heart Alliance, Munich, Germany

[5]Fraunhofer Institute for Digital Medicine MEVIS, Bremen, Germany

[6]Institute for Clinical Epidemiology and Biometry, University of Würzburg, Würzburg, Germany

[7]Institute of Social Medicine, Epidemiology and Health Economics, Charité – Universitätsmedizin Berlin, Berlin, Germany

[8]State Institute of Health, Bavarian Health and Food Safety Authority, Erlangen, Germany

[9]Institute for Medical Informatics, Biometry and Epidemiology, University Hospital Essen, University of Duisburg-Essen, Essen, Germany

[10]Institute for Prevention and Cancer Epidemiology, Medical Center – University of Freiburg, Faculty of Medicine, University of Freiburg, Freiburg, Germany

[11]Institute for Community Medicine, University Medicine Greifswald, Greifswald, Germany

[12]Division of Clinical Epidemiology and Aging Research, German Cancer Research Center (DKFZ), Heidelberg, Germany

[13]Division of Preventive Oncology, German Cancer Research Center (DKFZ) and National Center for Tumor Diseases (NCT), Heidelberg, Germany

[14]Department of Diagnostic and Interventional Radiology, University Hospital Augsburg, University of Augsburg, Germany

[15]Max-Delbrueck-Center for Molecular Medicine in the Helmholtz Association (MDC), Molecular Epidemiology Research Group, Berlin, Germany

[16]Max-Delbrueck-Center for Molecular Medicine in the Helmholtz Association (MDC), Biobank Technology Platform, Berlin, Germany

[17]Charité – Universitätsmedizin Berlin, Corporate Member of Freie Universität Berlin and Humboldt-Universität zu Berlin, Berlin, Germany

[18]Berlin Ultrahigh Field Facility (B.U.F.F.), Max Delbrück Center for Molecular Medicine in the Helmholtz Association (MDC), Berlin, Germany

[19]German Centre for Cardiovascular Research (DZHK), Partner Site Berlin, Berlin, Germany

[20]Department of Cardiology and Nephrology, HELIOS Hospital Berlin-Buch, Berlin, Germany

[21]Department of Diagnostic and Interventional Radiology and Neuroradiology, University Hospital Essen, University of Duisburg-Essen, Essen, Germany

[22]German Centre for Cardiovascular Research (DZHK), Partner Site Greifswald, University Medicine Greifswald, Greifswald, Germany

[23]Institute of Diagnostic Radiology and Neuroradiology, University Medicine Greifswald, Greifswald, Germany

[24]Division of Medical Physics, Department of Diagnostic and Interventional Radiology, Medical Center – University of Freiburg, Faculty of Medicine, University of Freiburg, Freiburg, Germany

[25]Department of Diagnostic and Interventional Radiology, Heidelberg University Hospital, Heidelberg, Germany

SUPPLEMENTAL MATERIAL

Table S1. Criteria catalog for visual image quality ratings in the NAKO MRI study (translated from version 02 of the internal documentation, example images omitted). Scores were assigned according to a 3-point Likert scale: 1) 'excellent' image quality not impaired by artifacts, images appropriate for data post-processing [marked below as Green]; 2) 'good' image quality with limited impairment by artifacts, images appropriate for data post-processing [marked below as Yellow]; 3) 'poor' image quality due to artifacts or insufficient coverage, images generally not appropriate for post-processing [marked below as Red]. The protocols used for functional or quantitative imaging (Resting State EPI BOLD, MOLLI SAX, and Multiecho 3D VIBE) were not rated.

**Neurodegenerative Focus**	
T1w 3D MPRAGE	<u>Minimal coverage</u><br>Left-right: from ear to ear; dorso-ventral: entire brain (high parietal region: all layers up to the skull included) to the lower border of the cerebellum; rostro-caudal: entire brain from frontal to occipital pole.<br>• Red if only partially covered or not covered at all<br>• Yellow if narrowly covered<br>• Green if completely covered
	<u>Minimum differentiable structures</u><br>Gray/white matter throughout the brain and cerebellum in very good contrast to each other, basal ganglia/thalamus clearly distinguishable
	<u>Other Considerations</u><br>Axial coverage up to the foramen magnum desired but not mandatory - Yellow; also Yellow if motion artifacts are present (if yes, how severe?) possibly Red; in the presence of magnetic field inhomogeneities, Red if target structures like the cerebrum or cerebellum are affected
2D FLAIR	<u>Minimal coverage</u><br>Left-right: from ear to ear; dorso-ventral: entire brain (high parietal, all layers up to the skull included) to the lower border of the cerebellum; rostro-caudal: entire brain from frontal to occipital pole<br>• Red if only partially covered or not covered at all<br>• Yellow if narrowly covered<br>• Green if completely covered
	<u>Minimum differentiable structures</u><br>Ventricles well-contrasted from white matter, basal ganglia (especially Nucl. caudatus) recognizable
	<u>Other Considerations</u><br>Green if lesions in white matter are visible, Yellow if motion artifacts (if yes, how strong?) possibly Red; in the presence of magnetic field inhomogeneities, Red if target structures like the cerebrum or cerebellum are affected

Table S1. (continued)

Cardiovascular Focus	
MRA 3D SPACE STIR	Minimal coverage<br>From lung apex to diaphragm<br>• Red if only partially covered or not covered at all<br>• Yellow if narrowly covered<br>• Green if completely covered
	Minimum differentiable structures<br>Large thoracic vessels (pulmonary arteries and veins at lobe level as well as ascending, arch, and descending aorta)
	Other Considerations<br>--
Cine SSFP LAX	Minimal coverage<br>At least the 3 planes (2CV, 3CV, or 4CV) completely; at least 1 complete cardiac cycle<br>• Red if completely wrong orientation of planes (e.g., missing a cardiac chamber) or completely not captured<br>• Yellow if still consistent with the correct orientation of long-axis sections (e.g., LVOT in the 4CV slightly cut)<br>• Green if correctly captured
	Minimum differentiable structures<br>Myocardium can be differentiated
	Other Considerations<br>Pulsation artifacts or magnetic field inhomogeneity or banding artifacts: Red if target structures (left and right ventricles) are affected or Yellow if they are still distinguishable. Yellow is also given if the atria are not evaluable
Cine SSFP SAX	Minimal coverage<br>Heart: Base to apex depicted; at least 1 complete cardiac cycle<br>• Red if only partially covered or not covered at all<br>• Yellow if narrowly covered<br>• Green if completely covered
	Minimum differentiable structures<br>Right and left ventricular myocardium functionally differentiable
	Other Considerations<br>Pulsation artifacts or magnetic field inhomogeneity or banding artifacts: Red if target structures (left and right ventricles) are affected or Yellow if they are still distinguishable

Table S1. (continued)

**Thoracoabdominal Focus**	
T2w HASTE	Minimal coverage<br>Lung apex to both sides of the lower border of the kidneys<br>• Red if only partially covered or not covered at all<br>• Yellow if narrowly covered<br>• Green if completely covered<br>(Optimal coverage: Complete imaging from the shoulder girdle above the clavicle to the beginning of the pelvis (L5/S1).)
	Minimum differentiable structures<br>Lung parenchyma, right/left pulmonary artery, diaphragm, liver, portal vein, pancreas, spleen, splenic vein
	Other Considerations<br>B0 inhomogeneities? Breathing artifacts? Foldover artifacts?
T1w 3D VIBE Dixon	Minimal coverage<br>Lung apex to below the trochanter minor on both sides<br>• Red if not completely covered<br>• Yellow if narrowly covered or if the lung apex is covered in the last slice.<br>• Green if completely covered<br>(Optimal coverage: Complete imaging from the shoulder girdle above the clavicle to the middle of the femur bone.)
	Minimum differentiable structures<br>Liver, portal vein, spleen, splenic vein, pancreas, adrenal glands, kidneys, renal pelvis, renal vein, visceral fat
	Other Considerations<br>SWAP artifacts (swap of fat/water voxels).<br>• Red if in visceral target organs (lung, mediastinum, liver, pancreas, kidneys) or >20% of visceral/subcutaneous fat of the torso<br>• Yellow if in visceral non-target organs (e.g., bladder) or 1-20% of visceral/subcutaneous fat of the torso<br>• Green if, for example, in the area of the extremities<br>B0 inhomogeneities (if caused by ECG cables/electrodes, still green; otherwise, proceed as for SWAP artifacts)? Breathing artifacts? Foldover artifacts?

Table S1. (continued)

**Musculoskeletal Focus**	
PDw FS 3D SPACE	Minimal coverage<br>Complete sacroiliac joint space (important not to cut off cranially) down to the caudal edge of the trochanteric mass (major and minor). Latero-cranially, the anterior superior iliac spine should be visualized, and caudally, the ischial bones should be included<br>• Red if more than 25% of the sacroiliac joint space dorsally is not covered or if the trochanteric region is not fully included<br>• Yellow if the anterior superior iliac spine is not covered or if the sacroiliac joint space dorsally is not entirely covered but more than 75% is included<br>• Green if completely covered
	Minimum differentiable structures<br>Visible sacroiliac joint space through paracoronal reconstruction of the sacrum, hip joint space, hip joint cartilage, symphysis space, femoral offset, iliac arteries, femoral arteries, bladder, rectum, sacroiliac joint space
	Other Considerations<br>Foldover artifacts? Fat suppression complete?<br>• Red if no fat saturation is present or if fat saturation did not work in the target organs (e.g., around the sacroiliac joint)<br>• Yellow if fat saturation in non-target organs is inadequate (e.g., signal-rich bone marrow in the ischial bones)<br>• Green in the absence of artifacts but also if, for example, only subcutaneous fat tissue appears incompletely saturated
T2w 2D FSE (Cervical Spine)	Minimal coverage<br>C2 to C7<br>• Red if not completely covered in terms of the number of vertebrae<br>• Yellow if vertebrae are not completely captured laterally or if neuroforamina are not fully captured in scoliosis<br>• Green if completely covered
	Minimum differentiable structures<br>Longitudinal ligaments, interspinal ligaments, intervertebral disc spaces, neuroforamina on both sides and their contents, spinal cord, facet joint space or cartilage, dorsal muscle fascia, dorsal part of the thyroid cartilage, course of the vertebral artery from C2 to entry into the neurocranium
	Other Considerations<br>Red if ventral saturator overlaps vertebral structures, Yellow if dorsal subcutaneous fat is depicted inhomogeneously
T2w 2D FSE (Thoracic Spine)	Minimal coverage<br>T1 to T12<br>• Red if not completely covered in terms of the number of vertebrae<br>• Yellow if vertebrae are not completely covered laterally or if neuroforamina are not fully captured in scoliosis<br>• Green if completely covered

Table S1. (continued)

	Minimum differentiable structures<br>Longitudinal ligaments, interspinal ligaments, intervertebral disc spaces, neuroforamina T1-12 and their contents, spinal cord and conus, facet joint space or cartilage, dorsal muscle fascia
	Other Considerations<br>Ventral saturator must not overlap vertebral structures, is dorsal subcutaneous fat homogeneously depicted? Yellow if artifacts due to aortic pulsation are present (if yes, how strong?), possibly Red
T2w 2D FSE (Lumbar Spine)	Minimal coverage<br>L1 to S4 (preferably S5)<br>• Red if not completely covered in terms of the number of vertebrae<br>• Yellow if vertebrae are not completely covered laterally or if neuroforamina are not fully captured in scoliosis<br>• Green if completely covered
	Minimum differentiable structures<br>Longitudinal ligaments, interspinal ligaments, intervertebral disc spaces, neuroforamina L1-S1 and their contents, in addition to nerve roots S2-4, conus and caudal fibers, facet joint space or cartilage, dorsal muscle fascia, height of the aortic bifurcation, pre-sacral fat tissue
	Other Considerations<br>Red if ventral saturator overlaps vertebral structures, Yellow if dorsal subcutaneous fat is depicted inhomogeneously

Figure S1. ROC curves from regularized regression of the combined set of image quality parameters with the outcome ‘chosen vs. discarded acquisition’: **a** across all protocols on 1,000 bootstrap samples, **b** across all protocols on 1,000 bootstrap samples (excluding the parameter ‘specific SNR’ to minimize missing data), **c-m** for individual protocols (three protocols had an insufficient sample size for inclusion: Resting State EPI BOLD, PDw FS 3D SPACE, and T2w 2D FSE). AUC with 95% CI corresponds to mean AUC and respective percentiles from the distribution over all bootstrap samples. Left to right: LASSO regression, Elastic Net regression, ridge regression.

a All protocols

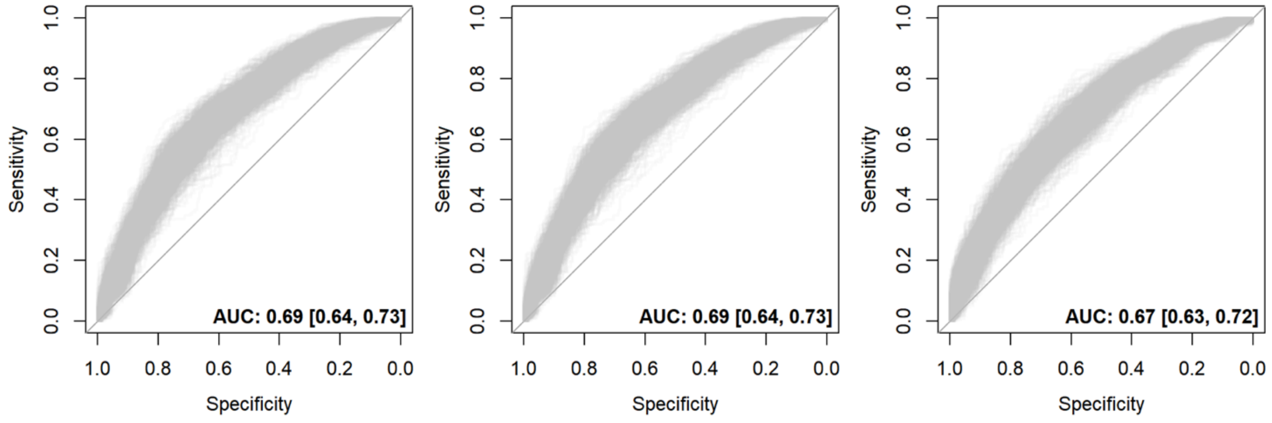

b All protocols ('specific SNR’ excluded)

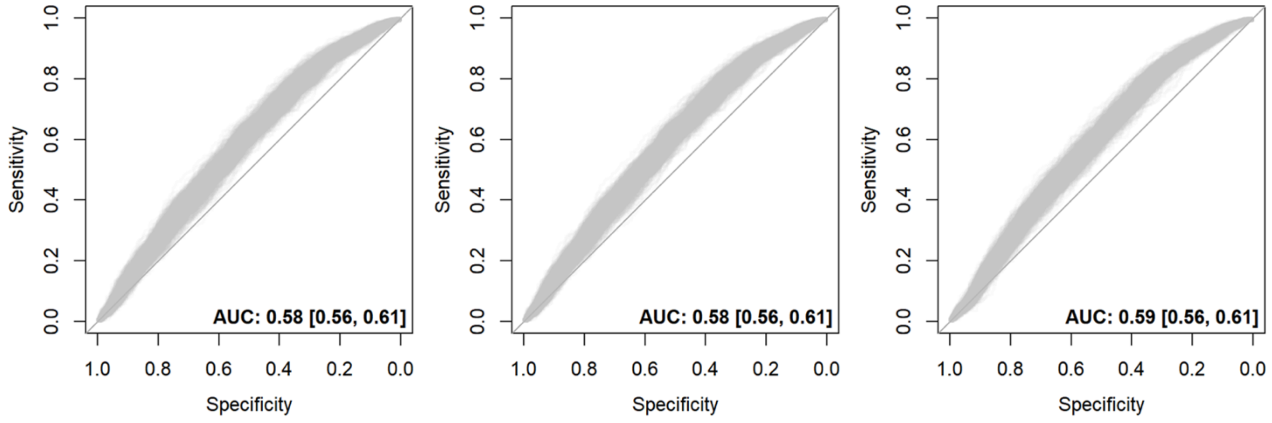

Figure S1. (continued)

c T1w 3D MPRAGE

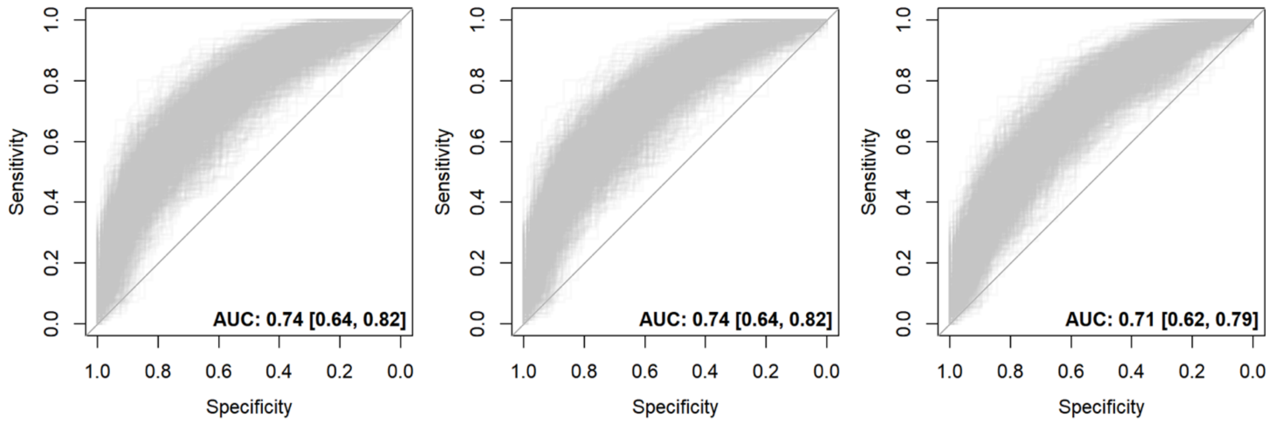

d 2D FLAIR

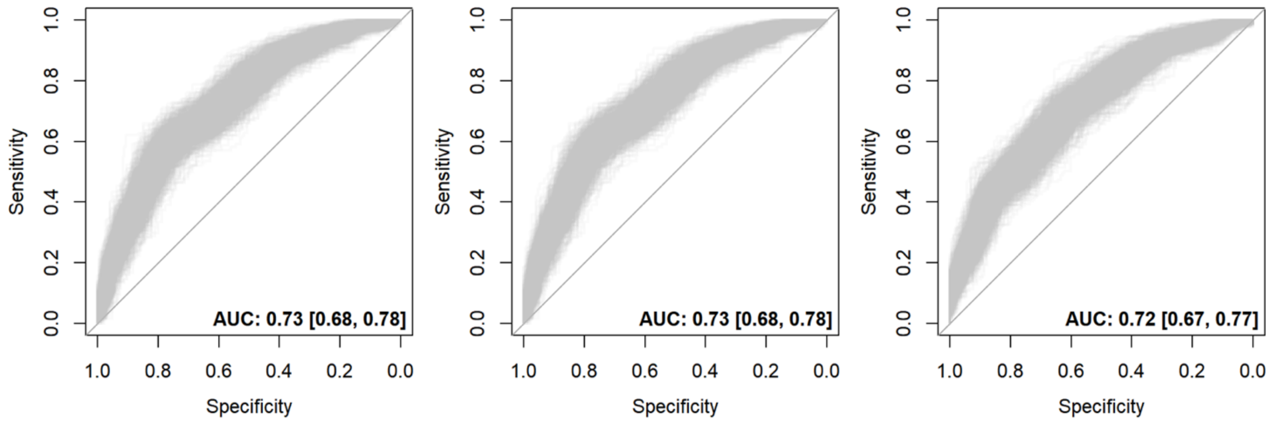

e MRA 3D SPACE STIR

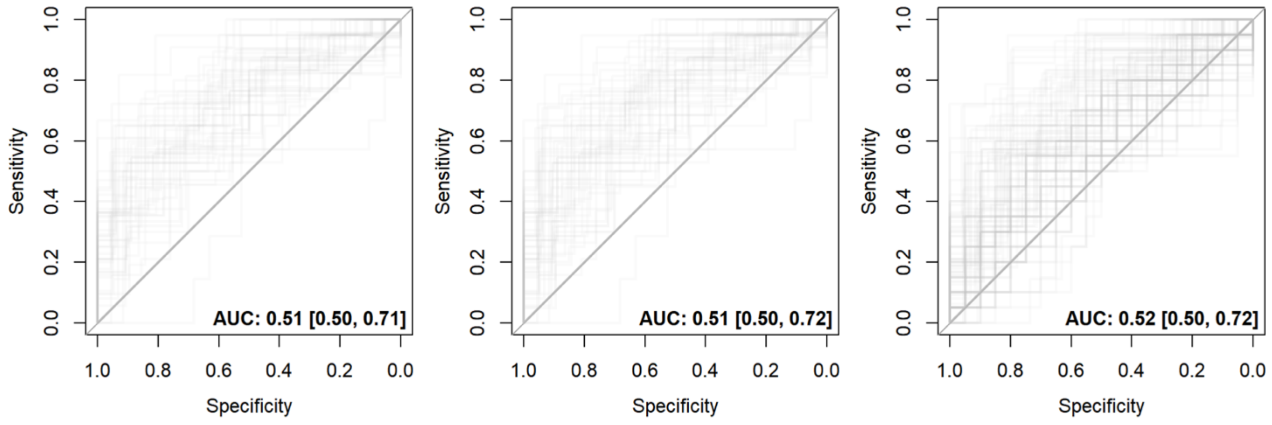

Figure S1. (continued)

f Cine SSFP LAX 2Ch

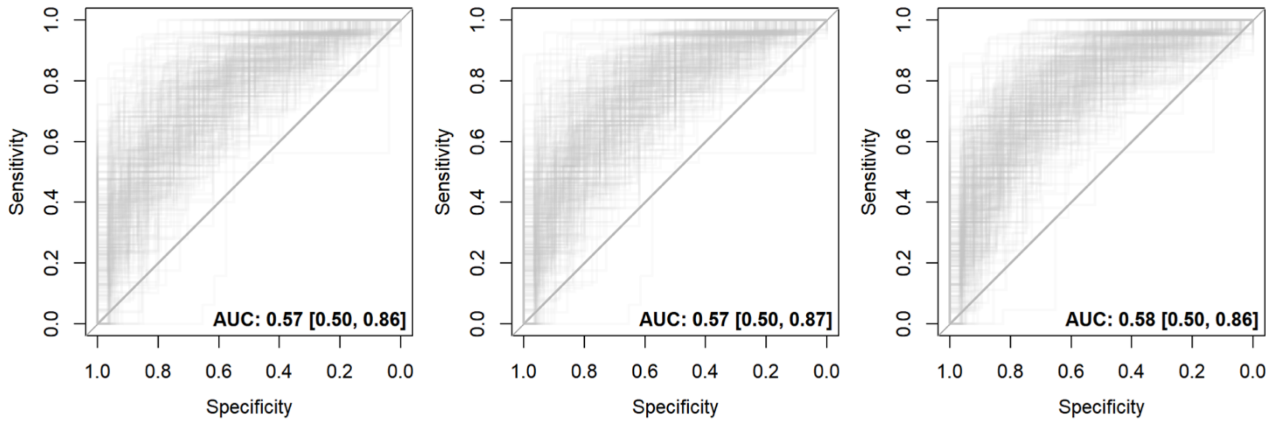

g Cine SSFP LAX 3Ch

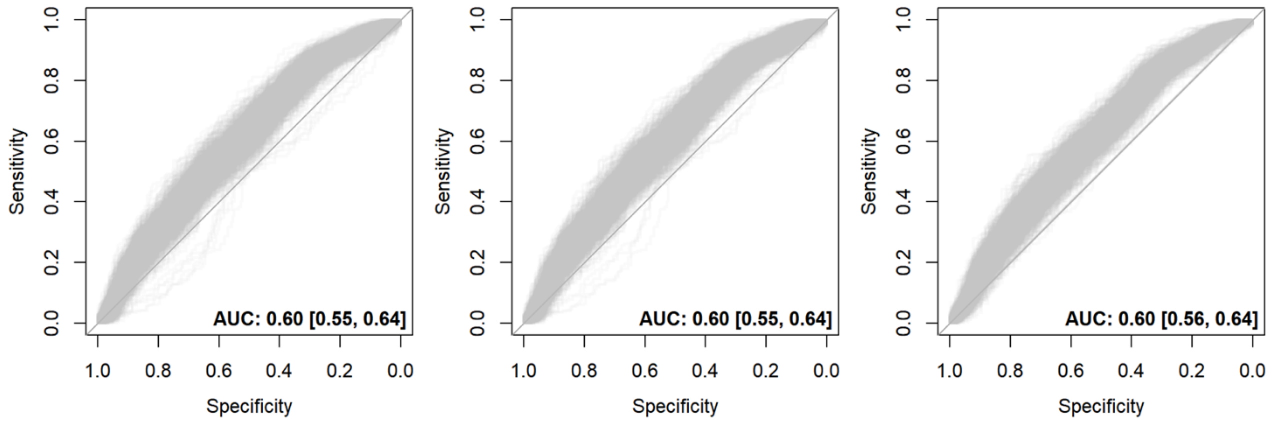

h Cine SSFP LAX 4Ch

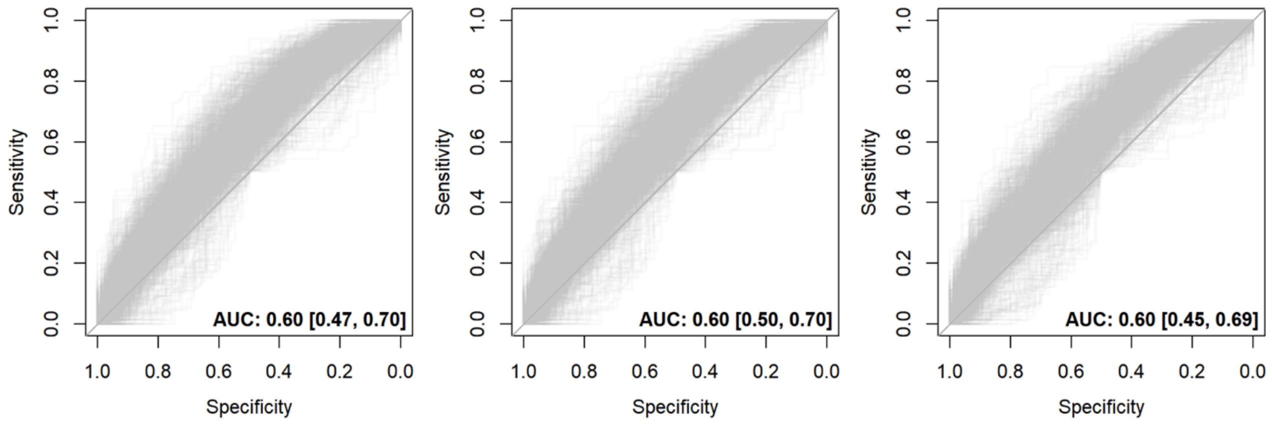

Figure S1. (continued)

i Cine SSFP SAX

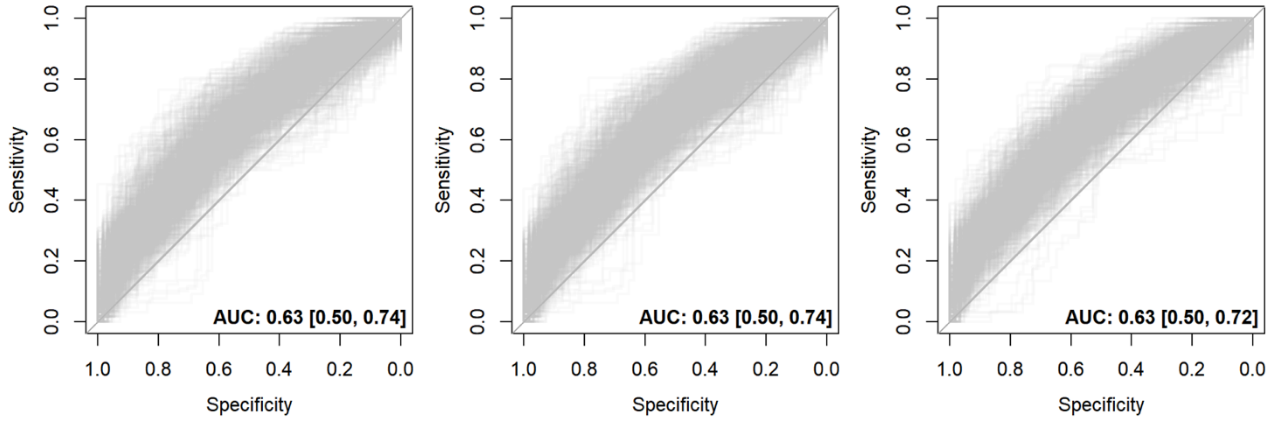

j MOLLI

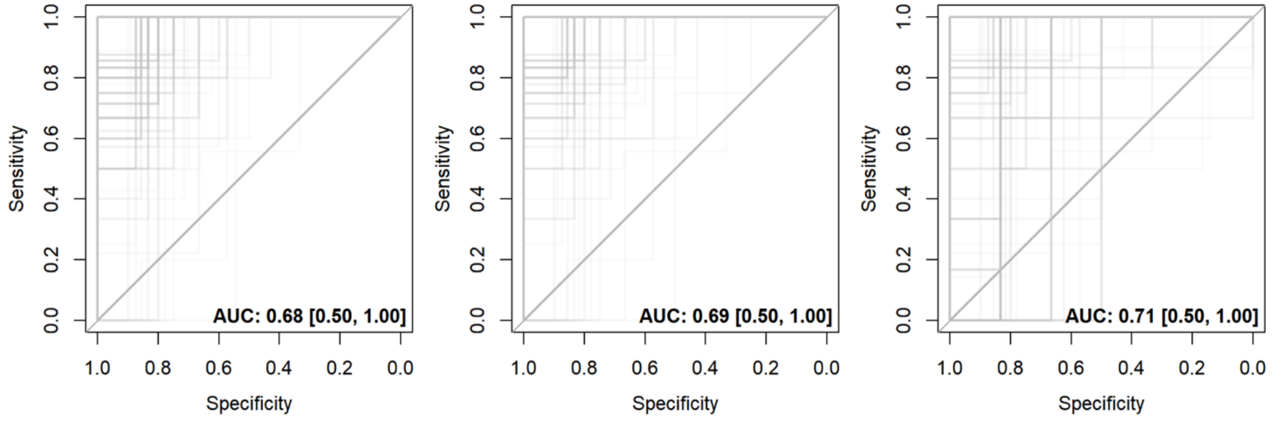

k T2w HASTE

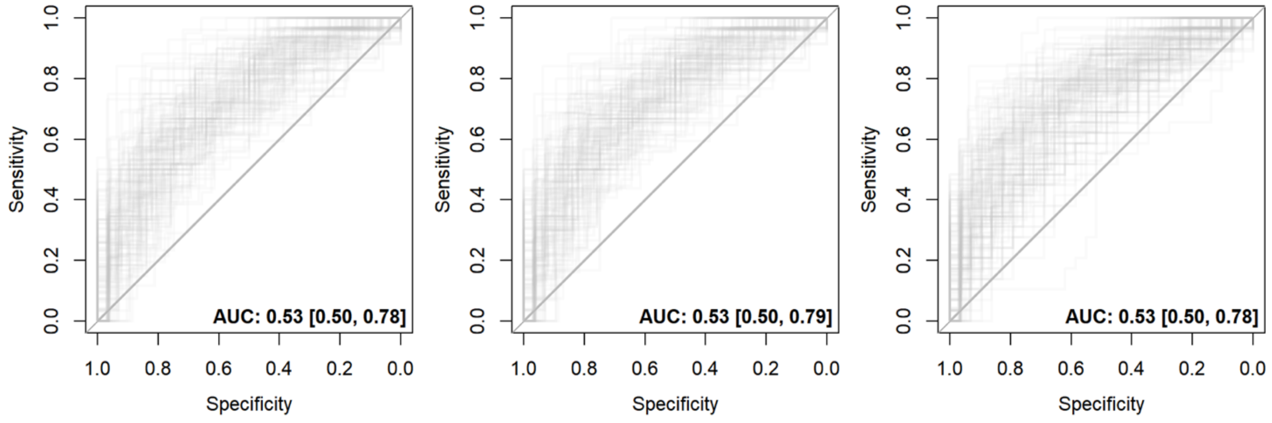

Figure S1. (continued)

l T1w 3D VIBE DIXON

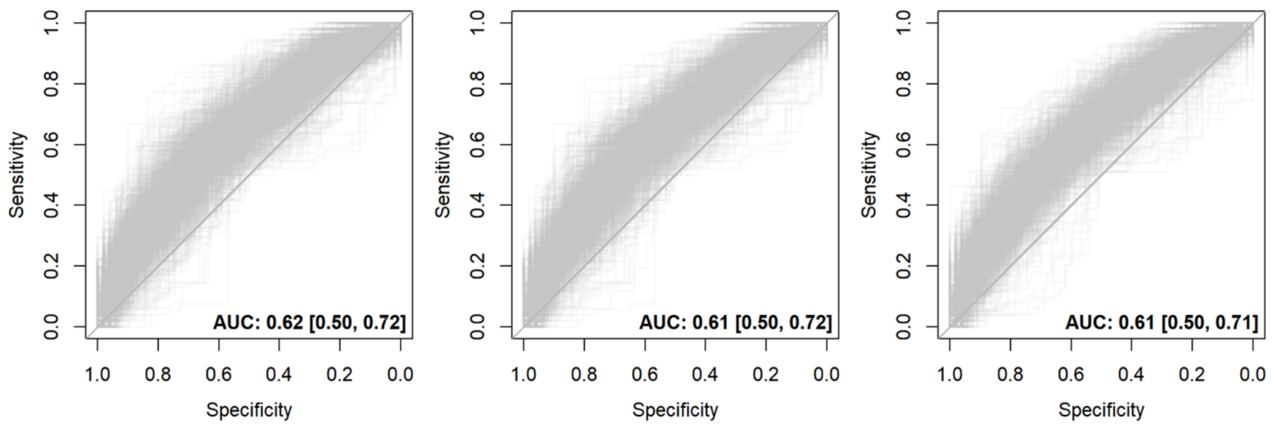

m Multiecho 3D VIBE

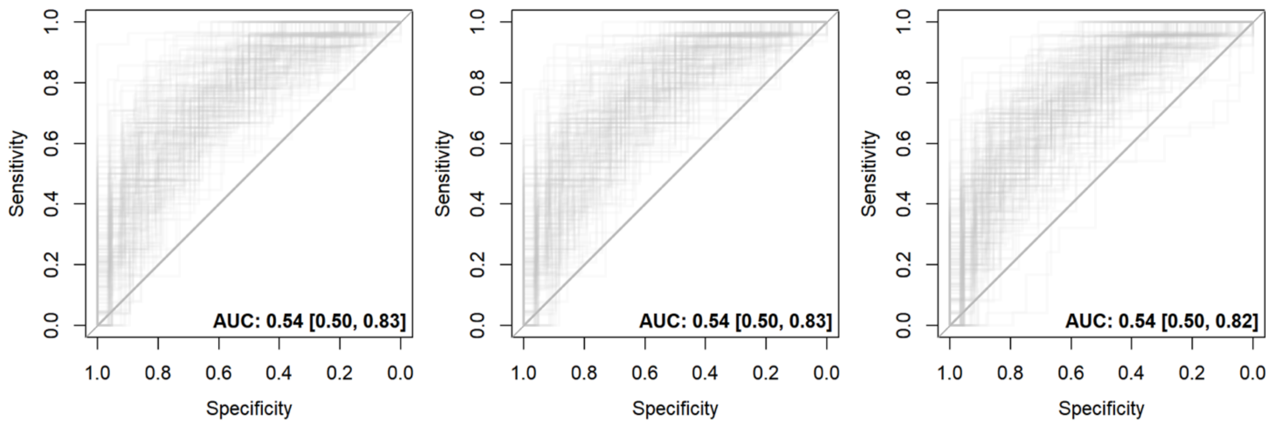

Figure S2. Variable selection frequencies from regularized regression with the outcome ‘chosen vs. discarded acquisition’: **a** across all protocols on 1,000 bootstrap samples, **b** across all protocols on 1,000 bootstrap samples (excluding the parameter ‘specific SNR’ to minimize missing data), **c-m** for individual protocols (three protocols had an insufficient sample size for inclusion: Resting State EPI BOLD, PDw FS 3D SPACE, and T2w 2D FSE). Left: LASSO regression, right: Elastic Net regression. As there is no variable selection in ridge regression, all selection frequencies are 100% (therefore not shown).

a All protocols

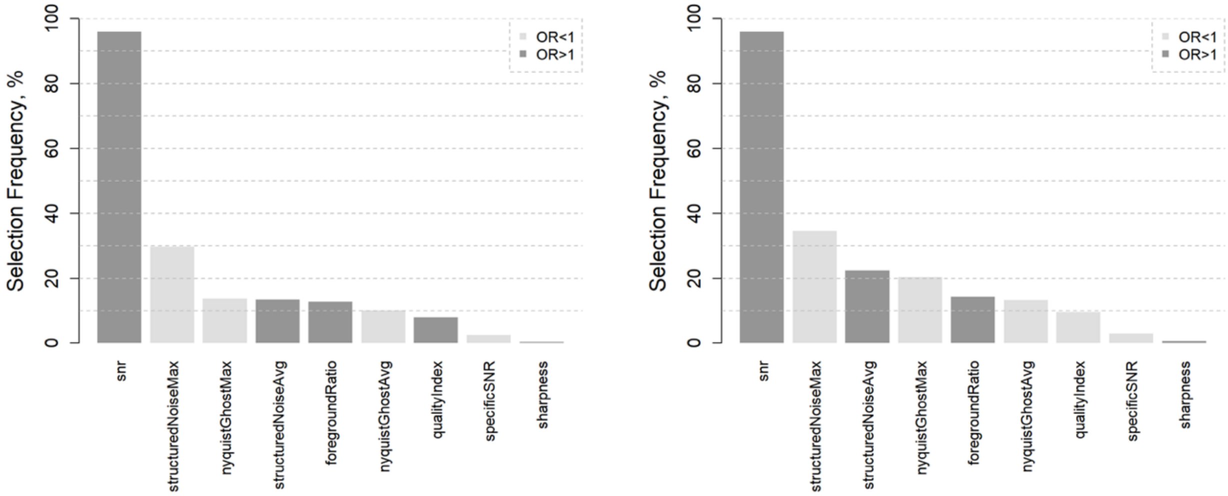

b All protocols ('specific SNR’ excluded)

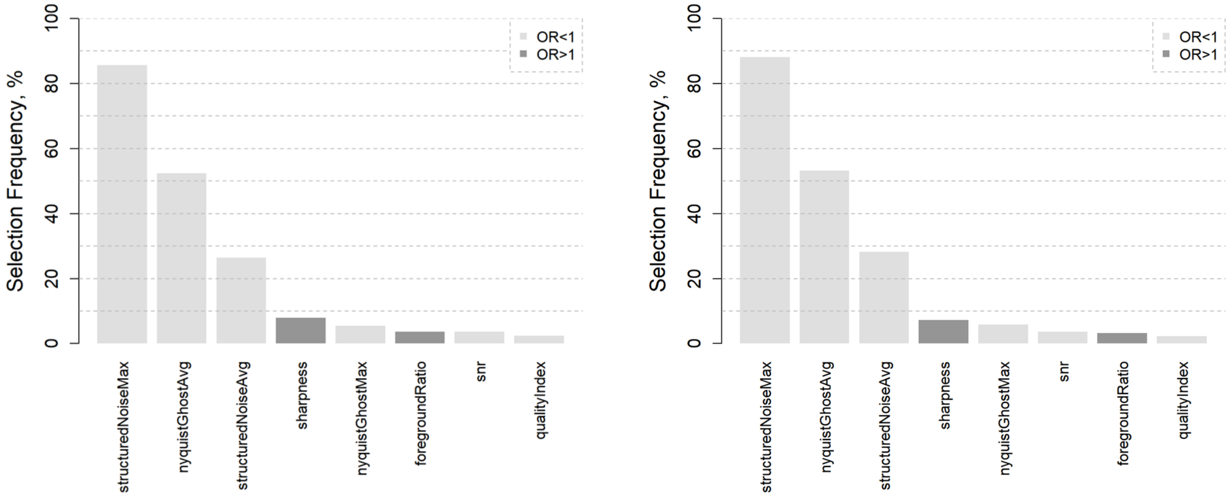

Figure S2. (continued)

c T1w 3D MPRAGE

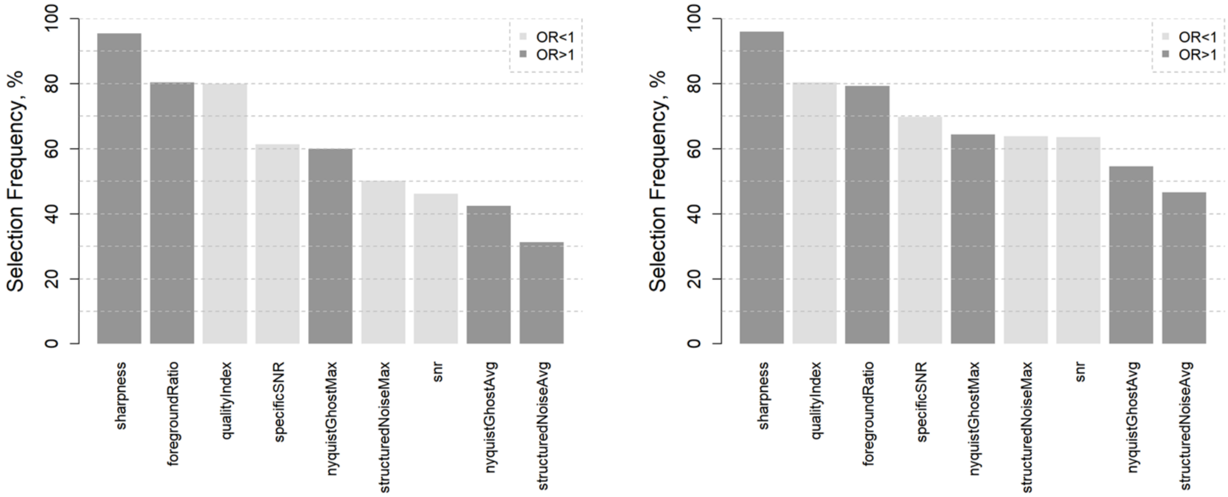

d 2D FLAIR

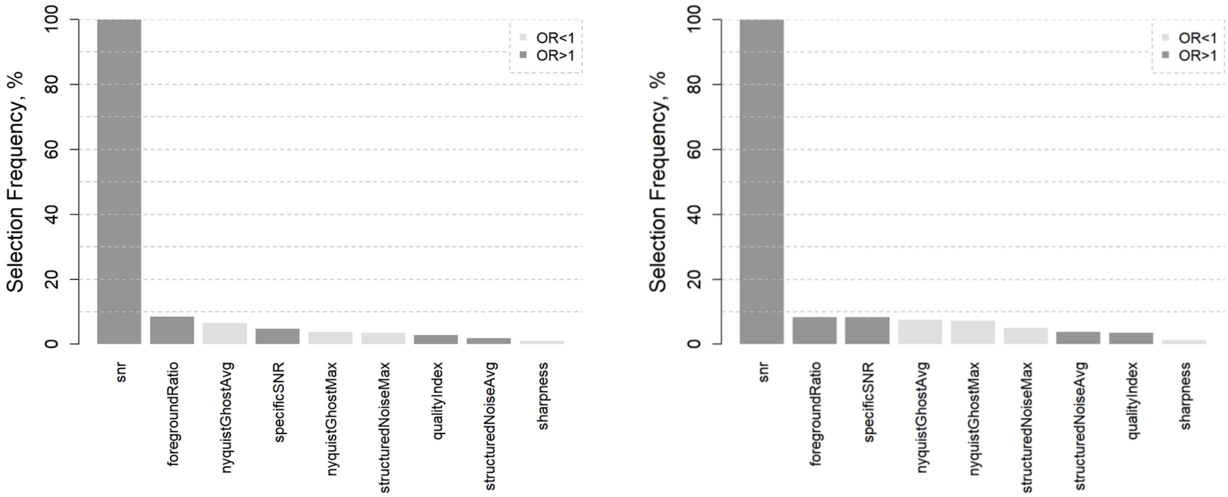

e MRA 3D SPACE STIR

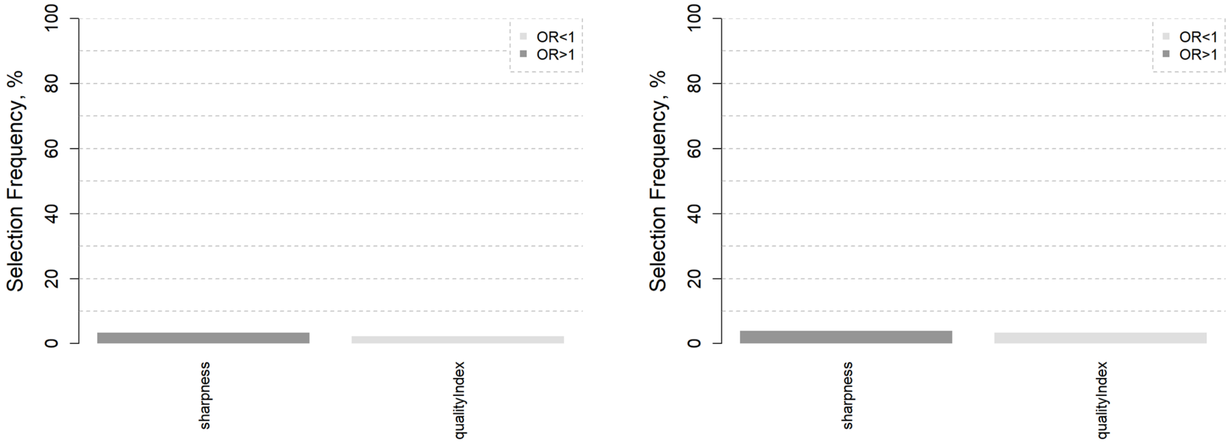

Figure S2. (continued)

f Cine SSFP LAX 2Ch

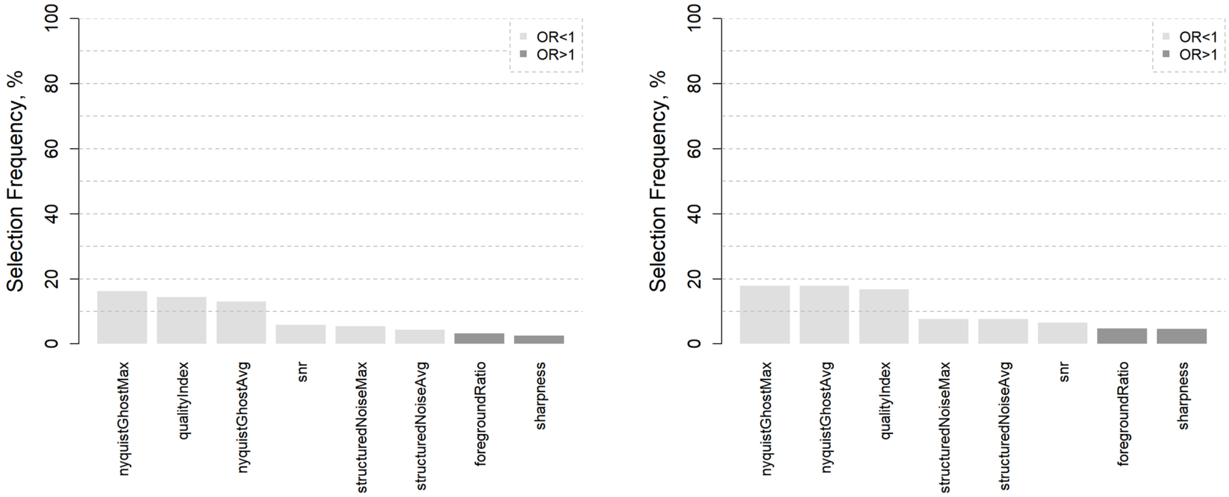

g Cine SSFP LAX 3Ch

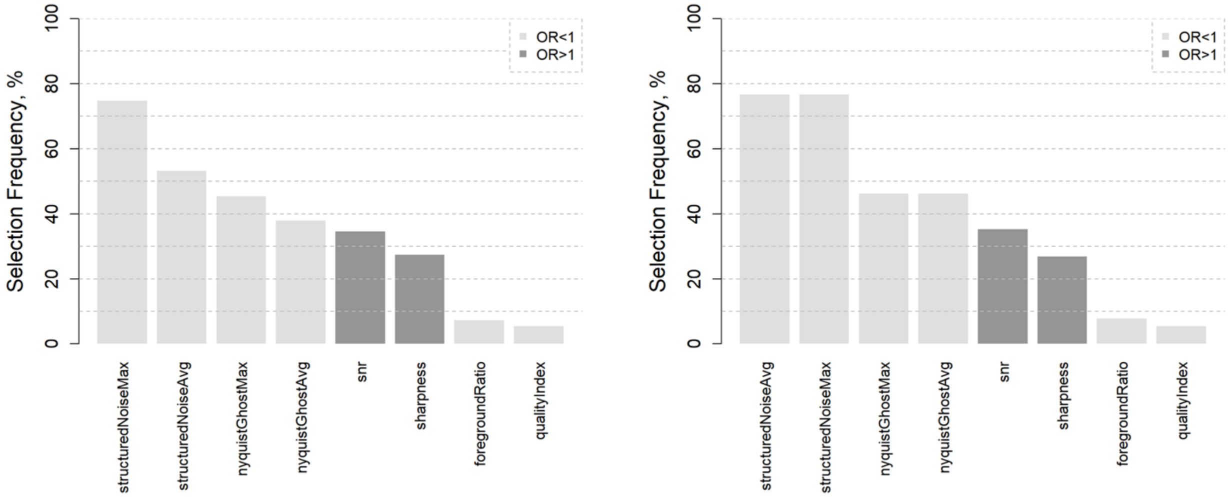

h Cine SSFP LAX 4Ch

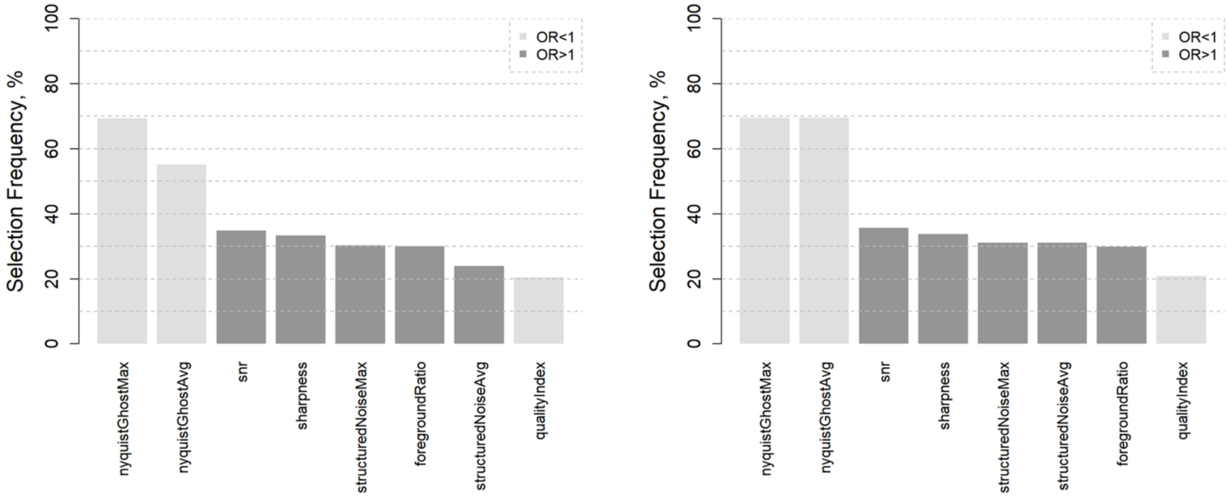

Figure S2. (continued)

i Cine SSFP SAX

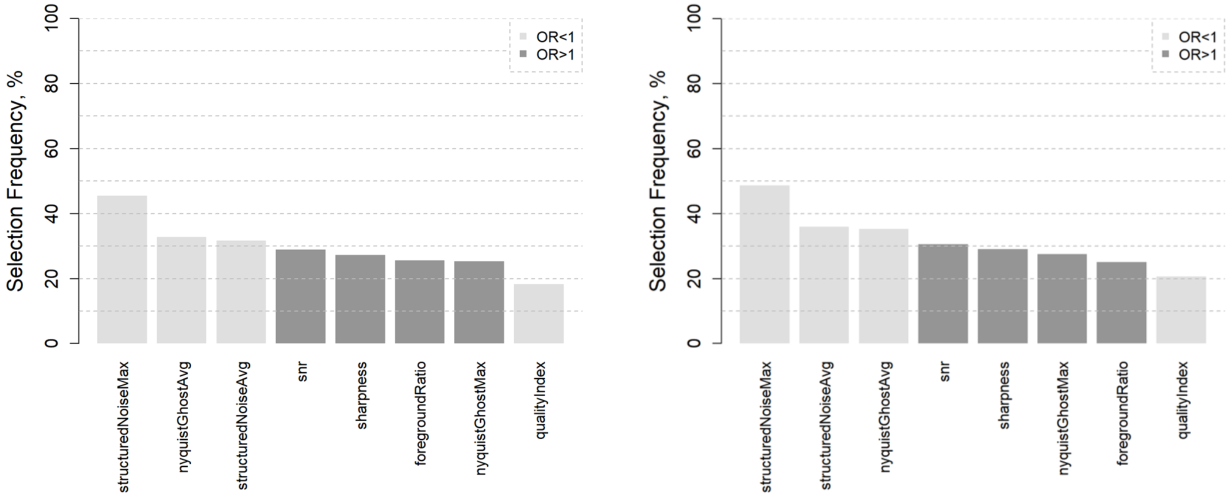

j MOLLI

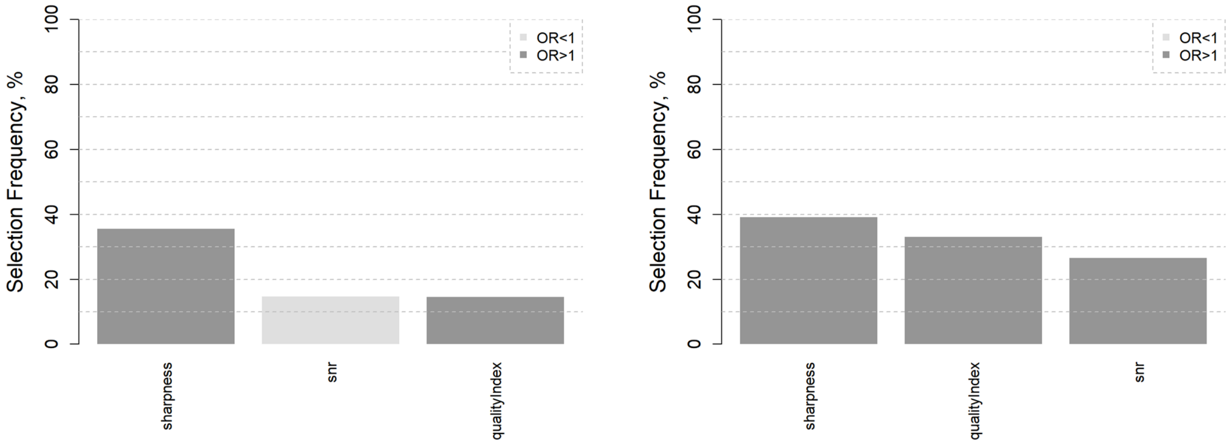

k T2w HASTE

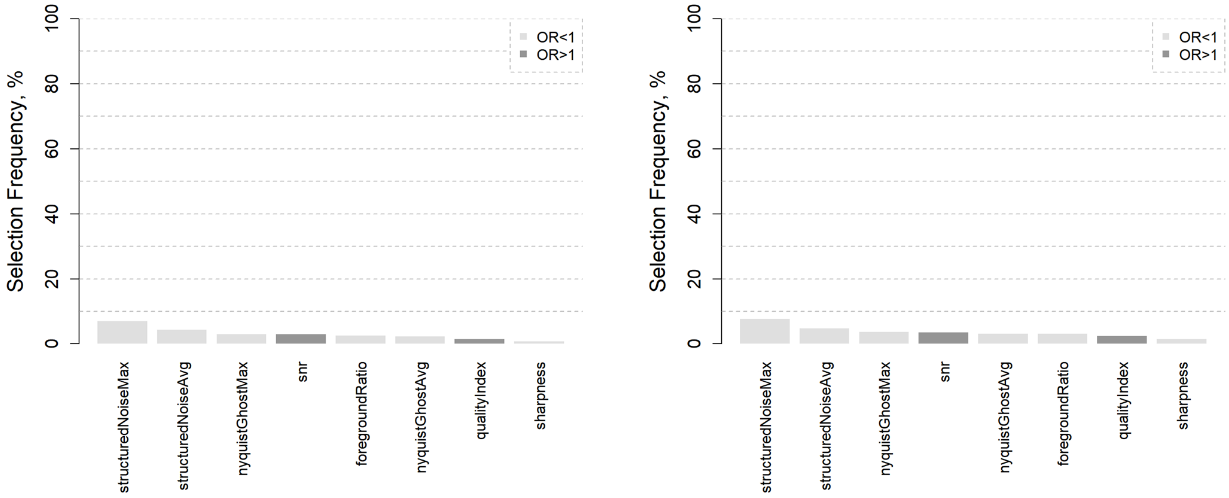

Figure S2. (continued)

l T1w 3D VIBE DIXON

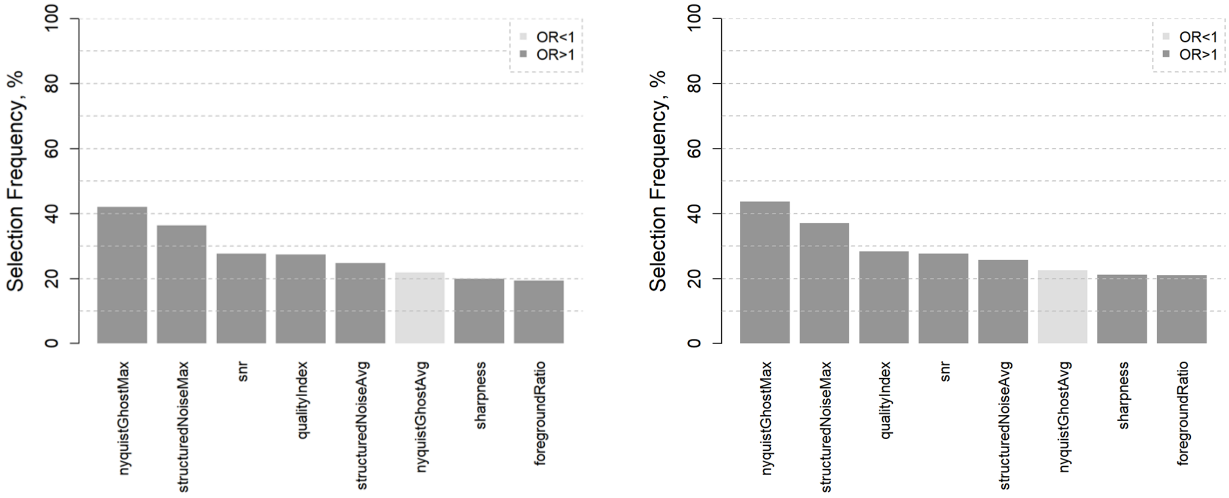

m Multiecho 3D VIBE

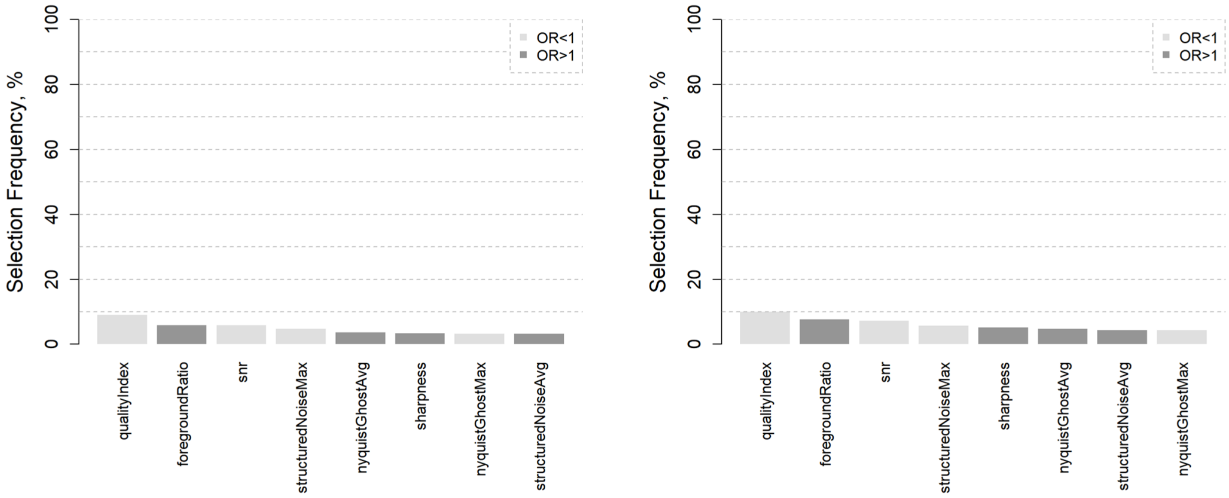

Supplement: Supplementary file 1 — Supplementary Information. [file 41598_2023_49569_MOESM1_ESM.pdf]
